# Massively multiplexed nucleic acid detection with Cas13

**DOI:** 10.1038/s41586-020-2279-8

**Published:** 2020-04-29

**Authors:** Cheri M. Ackerman, Cameron Myhrvold, Sri Gowtham Thakku, Catherine A. Freije, Hayden C. Metsky, David K. Yang, Simon H. Ye, Chloe K. Boehm, Tinna-Sólveig F. Kosoko-Thoroddsen, Jared Kehe, Tien G. Nguyen, Amber Carter, Anthony Kulesa, John R. Barnes, Vivien G. Dugan, Deborah T. Hung, Paul C. Blainey, Pardis C. Sabeti

**Affiliations:** 1 Broad Institute of Massachusetts Institute of Technology and Harvard, Cambridge, MA USA; 2 Department of Biological Engineering, MIT, Cambridge, MA USA; 3 Department of Organismic and Evolutionary Biology, Harvard University, Cambridge, MA USA; 4 Division of Health Sciences and Technology, Harvard Medical School and MIT, Cambridge, MA USA; 5 Ph.D. Program in Virology, Division of Medical Sciences, Harvard Medical School, Boston, MA USA; 6 Department of Electrical Engineering and Computer Science, MIT, Cambridge, MA USA; 7 Influenza Division, Centers for Disease Control and Prevention, Atlanta, GA USA; 8 Molecular Biology Department and Center for Computational and Integrative Biology, Massachusetts General Hospital, Boston, MA USA; 9 Koch Institute for Integrative Cancer Research at MIT, Cambridge, MA USA; 10 Howard Hughes Medical Institute, Chevy Chase, MD USA; 11 Department of Immunology and Infectious Disease, Harvard T.H. Chan School of Public Health, Boston, MA USA

**Keywords:** Biological techniques, Biotechnology, Viral infection

## Abstract

The great majority of globally circulating pathogens go undetected, undermining patient care and hindering outbreak preparedness and response. To enable routine surveillance and comprehensive diagnostic applications, there is a need for detection technologies that can scale to test many samples^1–3^ while simultaneously testing for many pathogens^4–6^. Here, we develop Combinatorial Arrayed Reactions for Multiplexed Evaluation of Nucleic acids (CARMEN), a platform for scalable, multiplexed pathogen detection. In the CARMEN platform, nanolitre droplets containing CRISPR-based nucleic acid detection reagents^7^ self-organize in a microwell array^8^ to pair with droplets of amplified samples, testing each sample against each CRISPR RNA (crRNA) in replicate. The combination of CARMEN and Cas13 detection (CARMEN–Cas13) enables robust testing of more than 4,500 crRNA–target pairs on a single array. Using CARMEN–Cas13, we developed a multiplexed assay that simultaneously differentiates all 169 human-associated viruses with at least 10 published genome sequences and rapidly incorporated an additional crRNA to detect the causative agent of the 2020 COVID-19 pandemic. CARMEN–Cas13 further enables comprehensive subtyping of influenza A strains and multiplexed identification of dozens of HIV drug-resistance mutations. The intrinsic multiplexing and throughput capabilities of CARMEN make it practical to scale, as miniaturization decreases reagent cost per test by more than 300-fold. Scalable, highly multiplexed CRISPR-based nucleic acid detection shifts diagnostic and surveillance efforts from targeted testing of high-priority samples to comprehensive testing of large sample sets, greatly benefiting patients and public health^9–11^.

## Abstract (web summary)

CRISPR-based nucleic acid detection is used in a platform that can simultaneously detect 169 human-associated viruses in multiple samples, providing scalable, multiplexed pathogen detection aimed at routine surveillance for public health.

## Main

**Fig. 1:**
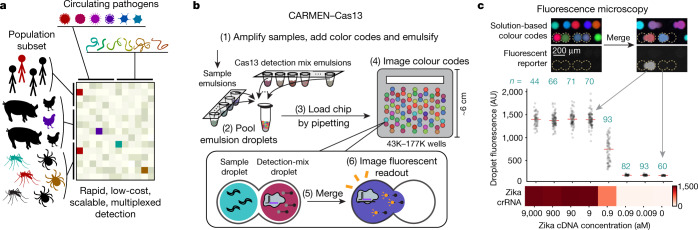
CARMEN–Cas13 achieves attomolar sensitivity. **a**, Identification of multiple circulating pathogens in human and animal populations represents a large-scale detection problem. **b**, Schematic of CARMEN–Cas13 workflow. **c**, Zika cDNA is detected by a single CARMEN–Cas13 assay with attomolar sensitivity and tens of replicate droplet pairs (black dots, numbers of replicates are in blue); red lines show medians and are used to construct the heat map below. Top, representative droplet images. AU, arbitrary units.

Infectious diseases are some of the greatest threats to human health and global security, yet there is no broadly available molecular test for the vast majority of disease-causing microbes, limiting their diagnosis and surveillance. Of the many viral species capable of infecting humans (576 of which had been sequenced and 169 of which had at least 10 published genomes^12^ by October 2018), only 39 had diagnostics approved by the FDA (US Food and Drug Administration; https://www.fda.gov). While laboratory developed tests have been developed for clinical testing of diverse pathogens at specific facilities, these tests can have long turnaround times and are rarely multiplexed. Routine comprehensive diagnostic testing would provide a previously unavailable data stream to inform patients, healthcare workers and policy makers to suppress and mitigate outbreaks. However, these tools are not widely available owing to the lack of a scalable and multiplexed technology to quickly and inexpensively identify any circulating pathogen (Fig. 1a). Comprehensive disease detection by sequencing or microarray hybridization provides detailed information about pathogen genotypes and evolution, but is difficult to implement on a wide scale owing to the cost and logistical demands of sample preparation^4–6,13^. Rapid, low-cost detection methods, such as CRISPR-based approaches, antigen-based tests, PCR or loop-mediated isothermal amplification (LAMP), detect only one or a small number of pathogens in a given reaction^1–3,7,14–16^. Combining the strengths of these approaches, an ideal diagnostic and surveillance technology would be highly multiplexed and easily scale across hundreds of samples.

Miniaturized and self-organizing microfluidic technology enables massive multiplexing of biochemical and cellular assays^17–21^. We recently developed a microwell-array system that harnesses miniaturization and self-organization to perform comprehensive combinatorial experiments. In this system, the user prepares a collection of inputs as droplet emulsions, and the input droplets organize themselves in the wells of the array, creating all possible pairwise combinations in replicate without additional user effort or active instrumentation^8^. We envisioned that CRISPR-based nucleic acid detection could be integrated with the microwell-array system to test many amplified samples for many analytes in parallel.

**Extended Data Fig. 1:**
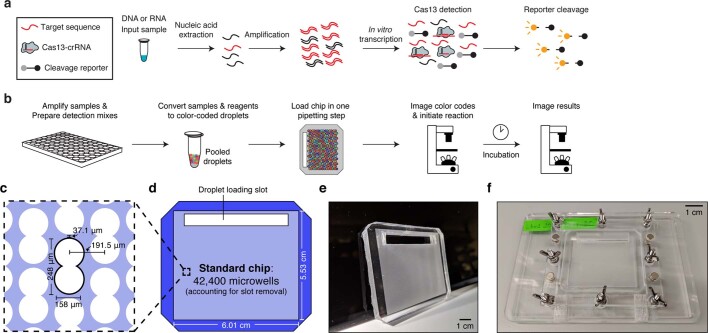
The CARMEN workflow at the molecular and macroscopic scale. **a**, Detailed molecular schematic of nucleic acid detection in CARMEN–Cas13. After amplification (with optional reverse transcription), detection is performed with Cas13, using in vitro transcription to convert amplified DNA into RNA. The resulting RNA is detected with exquisite sequence specificity by Cas13–crRNA complexes, and collateral cleavage activity of Cas13 produces a signal using a cleavage reporter RNA. **b**, Overview of the CARMEN workflow. Amplified samples and detection mixes are colour coded, emulsified and pooled into one tube. In a single pipetting step, the pool of droplets is loaded onto a chip, where the droplets self-organize into pairs. Fluorescence microscopy is used to read the colour code of each droplet, mapping the position of each sample and detection mix in the chip and droplets in each well are merged, initiating all reactions across the chip nearly instantaneously. After incubation, the reaction result for each well is read using fluorescence microscopy and mapped back to the colour codes of the sample and/or detection mix in each well. **c**, Microwell design optimized for droplets made from PCR products or detection mixes. **d**, Dimensions and layout of a standard chip. The area covered by the microwell array is shown in light blue. **e**, Photograph of a standard chip. **f**, Photograph of a standard chip sealed inside an acrylic loader, ready for imaging.

**Extended Data Fig. 2:**
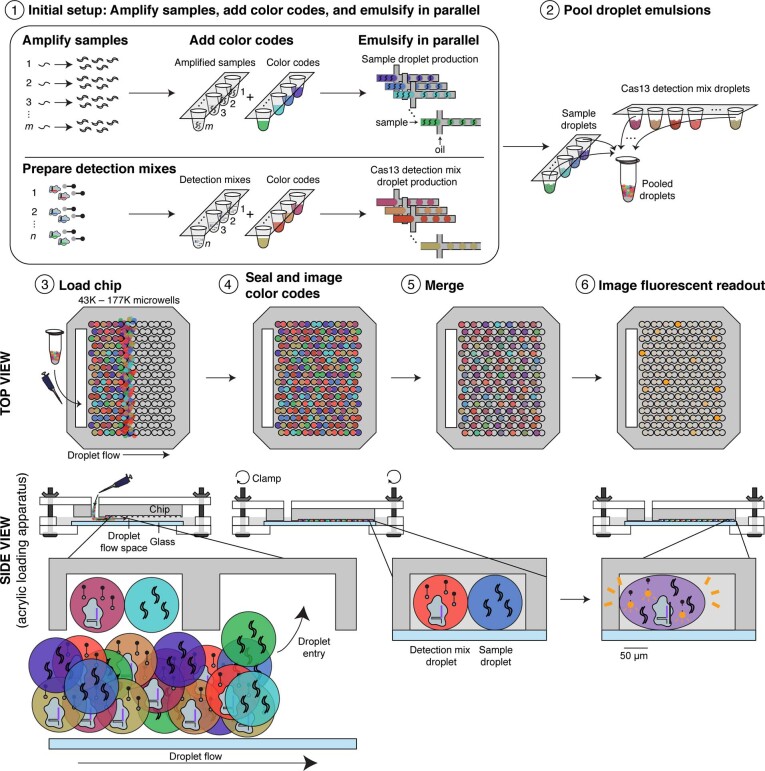
Detailed schematic of loader and chip function in CARMEN. Step 1, samples are amplified, colour coded and emulsified. In parallel, detection mixes are assembled, colour coded, and emulsified. Step 2, droplets from each emulsion are pooled into a single tube and mixed by pipetting. The pooling step is rapid to minimize small molecule exchange between droplets (see Supplementary Discussion 4). Step 3, the droplets are loaded into the chip in a single pipetting step. Side view, the droplets are deposited through the loading slot into the flow space between the chip and glass. Tilting the loader moves the pool of droplets around the flow space, allowing the droplets to float up into the microwells. Step 4, the chip is clamped against glass, isolating the contents of each microwell, and imaged by fluorescence microscopy to identify the colour code and position of each droplet. Step 5, droplets are merged, initiating the detection reaction. Step 6, the detection reactions in each microwell are monitored over time (a few minutes to 3 h) by fluorescence microscopy.

To enable highly multiplexed nucleic acid detection, we developed CARMEN (Fig. 1b, Extended Data Fig. 1). The inputs to CARMEN–Cas13 are samples that have been amplified by PCR or recombinase polymerase amplification (RPA) and Cas13-detection mixes, which contain Cas13, a sequence-specific CRISPR RNA (crRNA) and a cleavage reporter^7^ (Extended Data Fig. 1). Each amplified sample or detection mix is prepared in a conventional microtitre plate and combined with a distinct, solution-based fluorescent colour code that serves as an optical identifier. Each colour-coded solution is emulsified in fluorous oil to yield 1-nl droplets. Once emulsified, droplets from all samples and detection mixes are pooled into a single tube and—in one pipetting step—are loaded into a microwell-array chip moulded from polydimethylsiloxane (PDMS) (Fig. 1b, Extended Data Figs. 1, 2). Each microwell in the array accommodates two droplets from the pool at random, thereby spontaneously forming all pairwise combinations of dropletized inputs, and the array is sealed against a glass substrate to physically isolate each microwell. The contents of each microwell are determined by identifying the colour codes of the droplets using fluorescence microscopy. Exposure to an electric field merges the droplet pairs confined in each microwell and initiates all detection reactions simultaneously. Fluorescence microscopy is used to monitor each detection reaction (Fig. 1b, Extended Data Figs. 1, 2).

**Extended Data Fig. 3:**
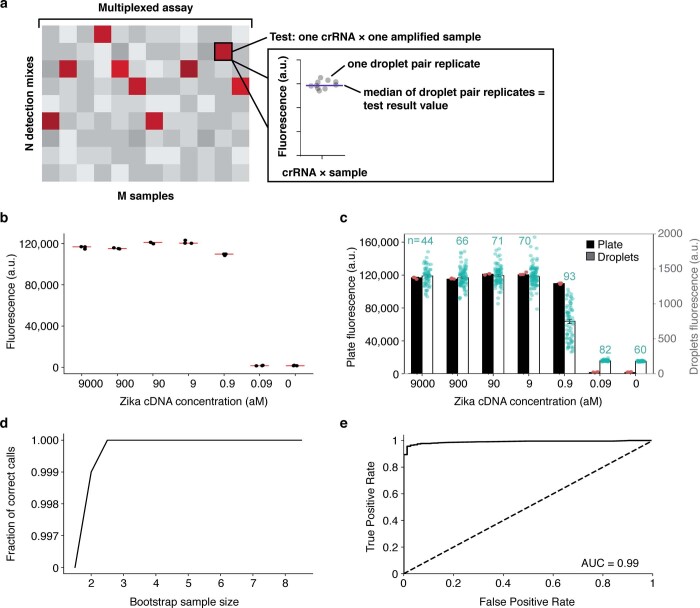
CARMEN multiplexed detection nomenclature and detection of Zika sequences. **a**, Assay, test and droplet-pair replicate nomenclature. Each multiplexed assay consists of a matrix of tests, where the dimensions of the matrix are *M* samples × *N* detection mixes. Each test is the result of one sample being evaluated by one detection mix, where the result of the test is the median value of a set of replicate droplet pairs in the microwell array. **b**, Plate reader data for SHERLOCK detection of synthetic Zika sequences at 3 h (*n* = 3 replicates). **c**, Comparison of plate reader and droplet (Fig. 1c) data. Replicates: *n* = 3 for plate reader data. Numbers of replicates for droplets data are indicated in teal. Error bars represent s.e.m. **d**, Bootstrap analysis of Zika detection in droplets. **e**, ROC curve for Zika detection in droplets.

CARMEN–Cas13 is sensitive, specific, and statistically robust. CARMEN–Cas13 detects Zika sequences with attomolar sensitivity, harnessing the collateral cleavage activity^22,23^ of CRISPR-Cas13 to match the sensitivity of specific high-sensitivity enzymatic reporter unlocking (SHERLOCK) and PCR-based assays^7,16^ (Fig. 1c, Extended Data Fig. 3, Supplementary Discussion 1). Additionally, CARMEN–Cas13 benefits from the specificity of SHERLOCK; sequence-specific identification is achieved through Cas13–crRNA binding and recognition, mitigating concerns about off-target amplification that are common in other nucleic acid detection methods (Supplementary Discussion 2). Each CARMEN–Cas13 assay combines *M* samples and *N* crRNAs to perform *M* × *N* tests, with each test comprising a set of crRNA–sample droplet pair replicates (Supplementary Discussion 3). The droplet-level CARMEN–Cas13 reactions are highly reproducible, enabling 1,000 tests per standard-capacity chip (Extended Data Fig. 3, Supplementary Discussion 4).

**Extended Data Fig. 4:**
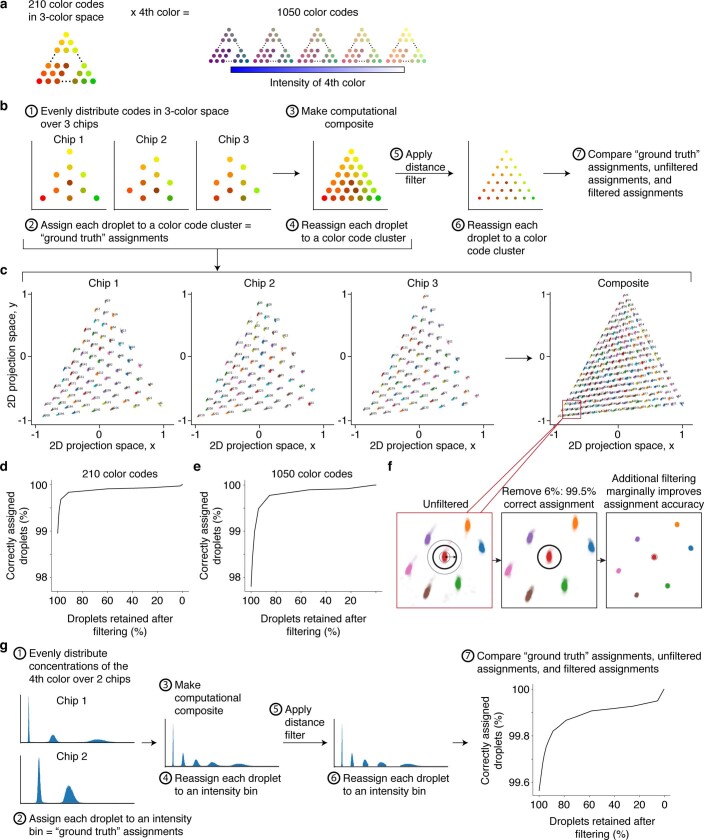
Design and characterization of 1,050 colour codes. **a**, Design of 1,050 colour codes. **b**, Schematic for characterization of 210 colour codes and the 3-colour dimension of 1,050 colour codes. **c**, Raw data from characterization of 210 colour codes. **d**, Performance of 210 colour codes in 3-colour space. **e**, Performance of 1,050 colour codes in 3-colour space. **f**, Illustration of the sliding distance filter (circle) in 3-colour space. **g**, Characterization schematic and performance of 1,050 colour codes in the 4th colour dimension.

**Extended Data Fig. 5:**
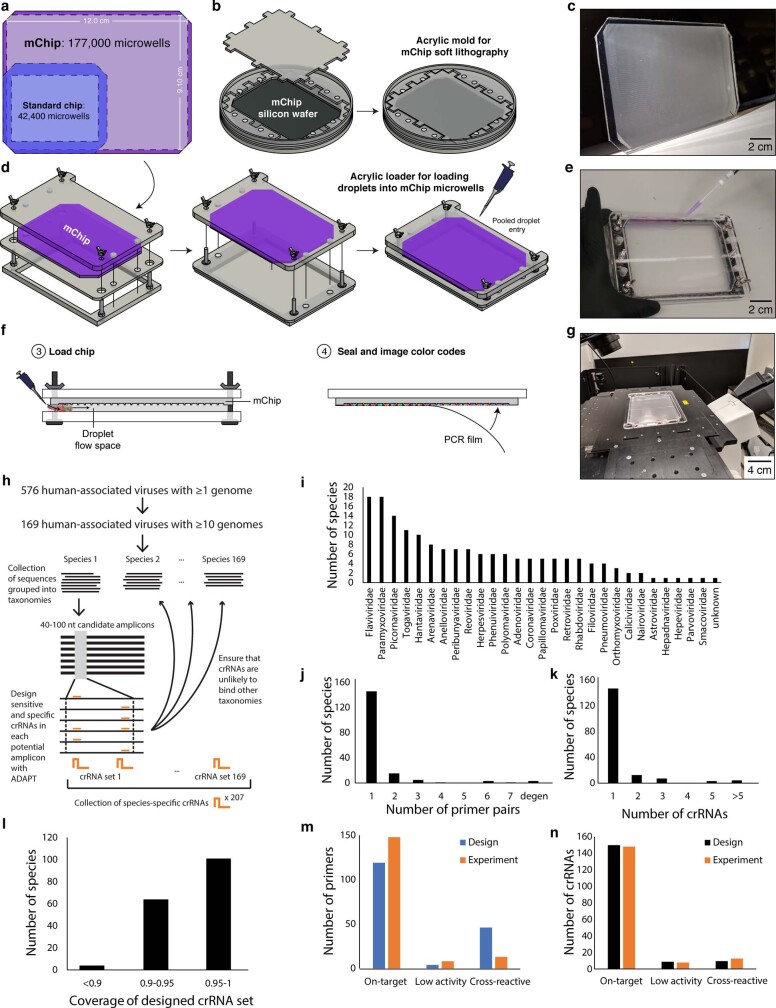
mChip and HV panel design schematic and statistics. **a**, Dimensions and layout of mChip, compared to a standard chip. The area covered by the microwell array is shown in purple. **b**, AutoCAD rendering of acrylic moulds used for mChip fabrication. **c**, Photograph of an mChip. **d**, Left, AutoCAD rendering of each part of the mChip loader; middle, AutoCAD rendering of the set-up of an mChip loader; right, AutoCAD rendering of an mChip in a loader, ready to be loaded. **e**, Photograph of an mChip being loaded. **f**, Loading and sealing mChip, corresponding to steps in Extended Data Fig. 2 (step 3, mChip loading). Droplets are deposited at the edge of the chip into the flow space between the chip and the acrylic loader. Tilting the loader moves the pool of droplets around the flow space, allowing the droplets to float up into the microwells. Step 4, the chip and loader lid are removed from the base and sealed against PCR film. No glass is used to seal the mChip. The sealed mChip, suspended from the acrylic loader lid, can be placed directly onto the microscope for imaging. **g**, Photograph of an mChip sealed and ready to be imaged. **h**, HV panel design. At the time we designed the panel (October 2018), there were 576 HV species with at least 1 genome neighbour in NCBI, and 169 with ≥10 genome neighbours. We aligned genomes by segment and analysed the sequence diversity using ADAPT to determine optimal primer and crRNA binding sites (see Methods, ‘HV panel design’ for details). **i**, Number of species in each family in the HV panel design. **j**, Number of primer pairs required to capture at least 90% of the sequence diversity within each species. Two species required the use of primer pairs containing degenerate bases. **k**, Number of crRNAs required to capture at least 90% of the sequence diversity within each species. **l**, The fraction of sequences within each species covered by each designed crRNA set; we were able to design small crRNA sets with 90% or greater coverage for 164 of the 169 species. **m**, **n**, To compare expected and observed performance for the HV panel, primers (**m**) and crRNAs (**n**) were classified into on-target, low activity or cross-reactive by sequence analysis (blue or black) or on the basis of experimental data (orange).

Accurate testing of multiple samples for hundreds of microbial pathogens requires higher throughput than is offered by existing multiplexed detection systems^2,24,25^. To enable highly multiplexed detection with high sample throughput, we developed a set of 1,050 solution-based colour codes using ratios of 4 commercially available, small-molecule fluorophores. Using the 1,050 colour codes, 99.5% of droplets can be correctly classified after permissive filtering that retains 94% of droplets (Extended Data Fig. 4, Supplementary Discussion 5). To match the throughput enabled by our 1,050 colour codes, we designed a massive-capacity chip (mChip) that allows more than 4,500 statistically replicated tests per chip (Extended Data Fig. 5). mChip reduces the reagent cost per test more than 300-fold relative to standard multiwell-plate SHERLOCK tests, while reducing pipetting steps and turnaround time (Extended Data Table 1, Supplementary Discussions 6, 7, 8).

**Fig. 2:**
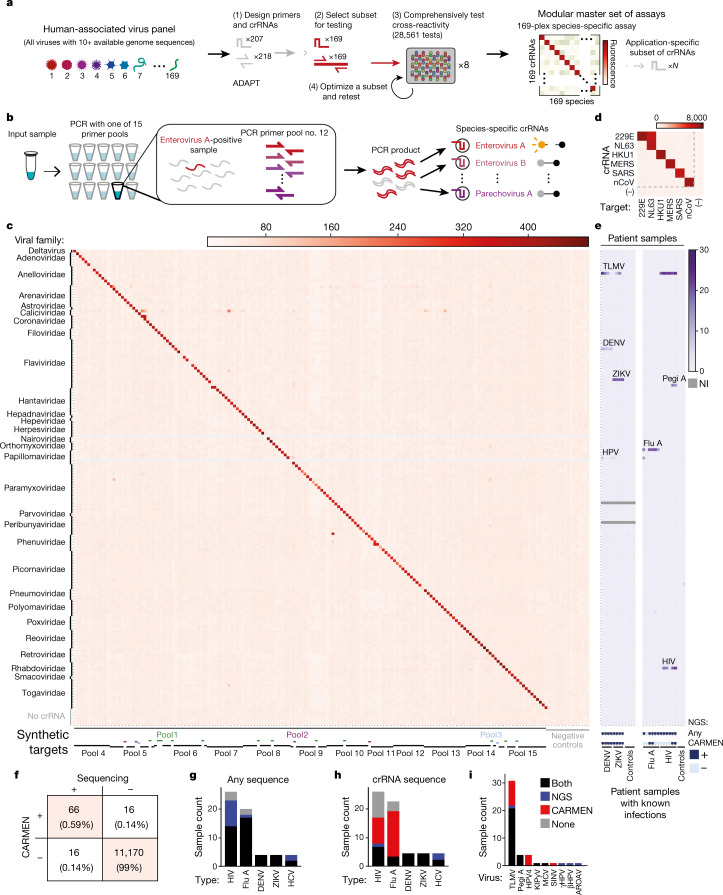
Comprehensive identification of HVs with CARMEN–Cas13. **a**, The development and testing of a panel for all 169 HVs with at least 10 available genome sequences. **b**, Experimental design using pooled PCR amplification. **c**, Testing a comprehensive HV panel with synthetic targets using CARMEN–Cas13. PCR primer pools 1–15 and viral families are below and to the left of the heat map, respectively. Grey lines, crRNAs not tested. **d**, Multiplexed coronavirus panel, comprising human coronaviruses 229E, NL63 and HKU1 Middle East respiratory syndrome (MERS) coronavirus; severe acute respiratory syndrome (SARS) coronavirus; novel coronavirus SARS-CoV-2 (nCoV); and negative control (−). **e**, Testing the HV panel with patient samples (additional data in Extended Data Fig. 8a). Heat maps indicate background-subtracted fluorescence after 1 h (**c**) or 30 min (**d**), or fold change over background (**e**). NI, not interpretable. **f**, Concordance of CARMEN and NGS in patient sample testing. Each box displays the number of tests and the percentage of the total. **g**, Identification by NGS or CARMEN of any viral sequence from the known infections in patient samples (for example, detection of HIV in samples from patients with HIV). **h**, Identification by NGS or CARMEN of the crRNA target for each known infection. **i**, Positive test results in patient samples for viruses other than the known infections. DENV, dengue virus; ZIKV, Zika virus; HCV, hepatitis C virus; TLMV, Torque teno-like mini virus; Pegi A, pegivirus A; HPV4, human papillomavirus 4; KIPyV, KI polyomavirus; MCV, Merkel cell polyomavirus; SINV, Sindbis virus; γHPV, gamma human papillomavirus; βHPV, beta human papillomavirus 2; AROAV, aroa virus. CARMEN does not test for γHPV or AROAV because fewer than 10 γHPV or AROAV genomes had been published before 24 October 2018.

We designed a CARMEN–Cas13 assay to selectively and simultaneously test dozens of samples for all 169 human-associated viruses (HVs) with at least 10 available published genomes (as of 24 October 2018). We applied ADAPT (see Methods, ‘HV panel design’) (Metsky et al., manuscript in preparation) to the published viral genomes of viruses represented in our HV panel to select amplicons for PCR-primer pools, using primer3 to optimize primer sequences^26^. ADAPT accepts a collection of sequences arranged into groups (for example, all known sequences within a species). For each group, ADAPT searches for an optimal set of crRNAs that are sensitive to the sequences within the group (that is, they detect a desired fraction of sequences) and are unlikely to detect sequences in the other groups (Extended Data Fig. 5h). We used ADAPT to design a small set of crRNA sequences for each species such that, accounting for genome diversity on NCBI GenBank, each crRNA set provides high coverage (more than 90% of sequences detected) within its targeted species and high selectivity against other species (Fig. 2a, Extended Data Fig. 5). We designed the HV panel as a modular master set of nucleic acid detection assays which can be customized by the end user for diverse applications (Fig. 2a).

**Extended Data Fig. 6:**
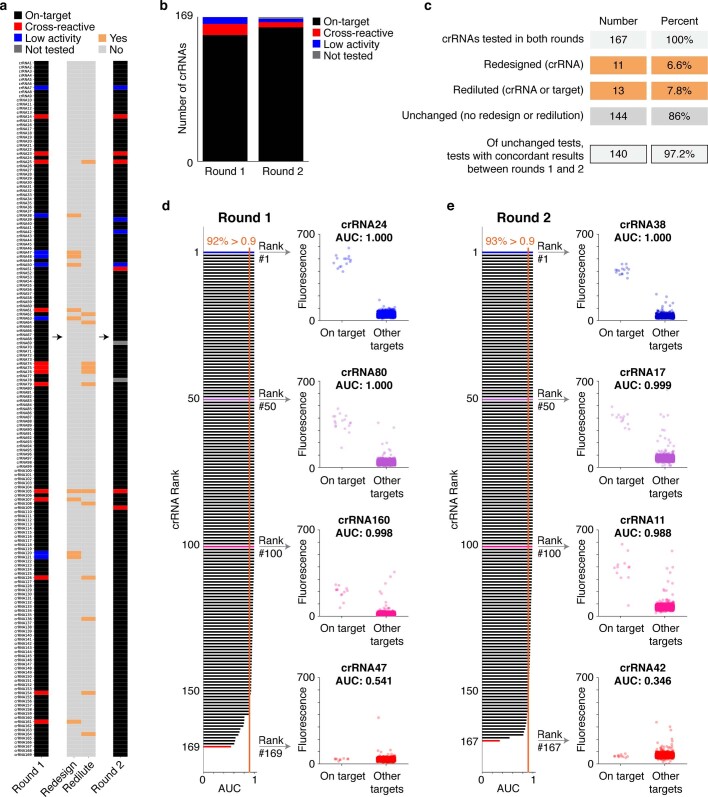
crRNA performance during HV panel testing. **a**, Individual guide performance in rounds 1 and 2. Redesign and re-dilution between rounds of testing are indicated between the data from rounds 1 and 2. On-target: reactivity above threshold for intended target only. Cross-reactive: off-target reactivity above threshold. Low activity: no reactivity above threshold. **b**, Summary bar graph of crRNA performance in rounds 1 and 2. **c**, Summary table of redesign, re-dilution and concordance between rounds 1 and 2 for unchanged tests. **d**, **e**, Round 1 (**d**) and round 2 (**e**) ranked AUCs for ROCs for on-target versus off-target reactivity in round 1 of testing. Representative on-target and off-target distributions are shown for the indicated ranks.

**Extended Data Fig. 7:**
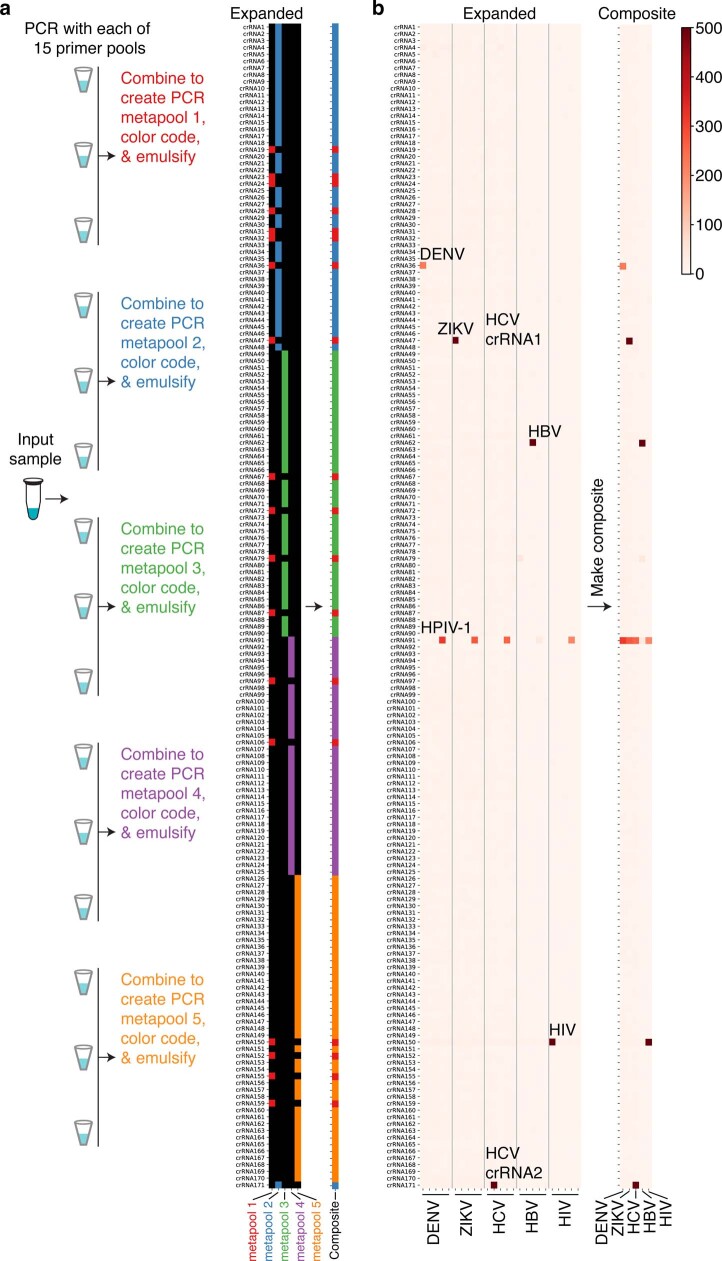
Synthetic target testing with HV panel. **a**, Sample handling and data analysis for unknown samples. Following multiplexed PCR with 15 pools, PCR products are combined into sets of 3 (PCR metapools). A subset of the crRNAs correspond to the primers in each PCR metapool, shown by the colours in the expanded heat map. Composite heat maps are generated by combining data from the metapools in the expanded heat map. **b**, Five synthetic targets (10^4^ copies per μl) were amplified with all primer pools and detected using 169 crRNAs from the HV panel plus HCV crRNA 2. The heat map indicates background-subtracted fluorescence after 1 h.

Taking advantage of the massive multiplexing capabilities of CARMEN–Cas13, we tested the full HV panel and demonstrated its performance. We computationally selected the optimal crRNA from each species set in the design (169 total, see Supplementary Discussion 9a) and evaluated each against synthetic consensus sequences for every species, which had each been amplified using their corresponding primer pool (184 total PCR products, including controls; Fig. 2b), for a total of 30,912 tests performed across 8 mChips (see Supplementary Table 1). We performed two rounds of testing, improving the designs for 11 species (6.5%) for the second round. We observed 97.2% concordance between the two rounds for unchanged designs, demonstrating that individual crRNAs can be improved without altering the performance of the rest of the assay (Extended Data Fig. 6, Supplementary Discussion 9, Supplementary Data 3). In round two, 157 of 167 (94%) of crRNAs were selective for their targets with signal above threshold (6 × s.d. above background), with a median area under the curve (AUC) of 0.997 across all 167 crRNAs (Extended Data Fig. 6). Furthermore, widespread cross-reactivity is not observed, even when synthetic targets are amplified with all primer pools (Extended Data Fig. 7).

As an outbreak of COVID-19 emerged during the manuscript review process, we rapidly incorporated a new test^27^ for the novel coronavirus SARS-CoV-2 into a coronavirus panel taken from the HV panel, demonstrating the power of this modular master set to be adapted to real-world challenges (Fig. 2d). Using a single mChip, more than 400 samples can be tested in parallel against our coronavirus panel.

**Fig. 3:**
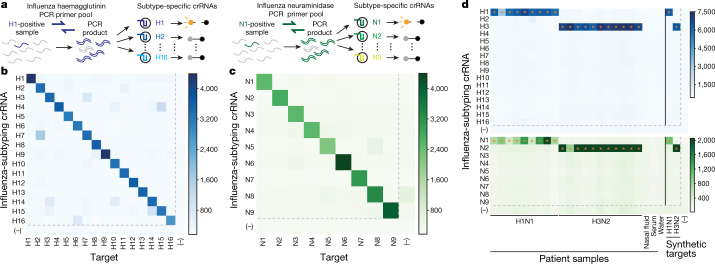
Influenza subtype discrimination with CARMEN–Cas13. **a**, Schematic of influenza A subtype discrimination using CARMEN–Cas13. **b**, Discrimination of H1–H16 using CARMEN–Cas13. **c**, Discrimination of N1–N9 using CARMEN–Cas13. **d**, Identification of H and N subtypes from patient samples and mixed synthetic targets (Methods, ‘Cas13 detection reactions’ under ‘General procedures’). Heat maps indicate background-subtracted fluorescence after 1 h (for H1–H16 discrimination) or 3 h (for N1–N9 discrimination) of Cas13 detection. In **b**–**d**, synthetic targets were used at 10^4^ copies per μl. Asterisks in **d** indicate a signal above threshold for a synthetic target or patient sample.

**Extended Data Fig. 8:**
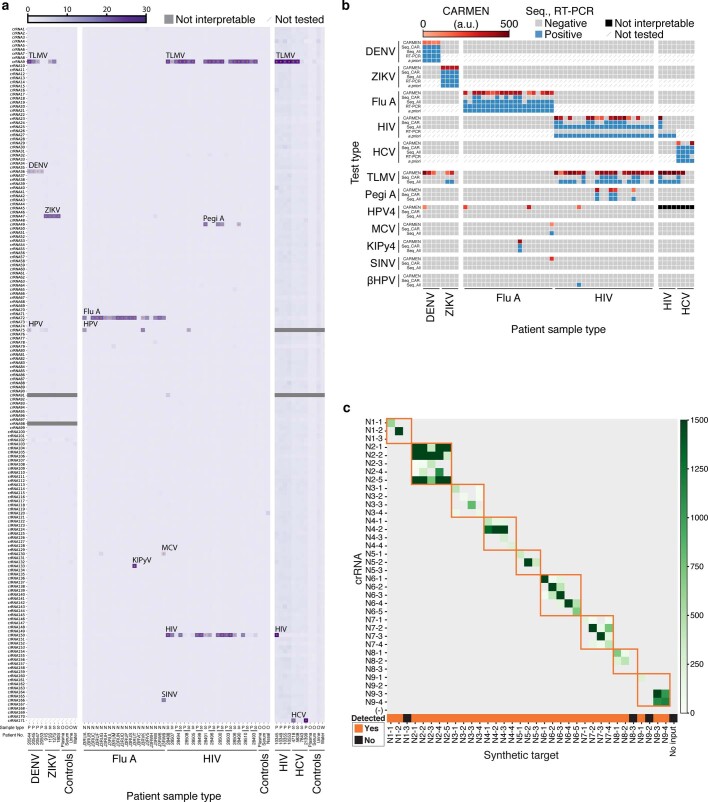
Testing of clinical samples with HV panel and performance of influenza A subtyping. **a**, CARMEN testing of patient samples and healthy pooled controls using the HV panel. Colour bar indicates fold change above background at 1 h for most crRNAs (3 h time point is shown for HIV and HCV crRNAs). Tests that could not be interpreted owing to the presence of signal above background in the negative controls are coloured in dark grey (not interpretable). Sample types: N, throat and nasal swabs; O, pooled healthy controls; P, plasma; S, serum; and W, water. Orange asterisks indicate signal above threshold (sixfold higher than background). **b**, Comparison of results from CARMEN, RNA sequencing-based identification of the sequence targeted by the indicated crRNA (Seq_CAR.), RNA sequencing-based identification of any sequences from the indicated virus (Seq_All), RT–PCR for the indicated virus, and a priori expectation based on information from the patient sample provider (a priori) for 4 dengue, 4 Zika, 20 influenza A, 26 HIV and 4 HCV patient samples. CARMEN testing was done over three rounds (as indicated by vertical separation between sections). Threshold cut-offs for making calls were: CARMEN, sixfold higher than background; Seq_CAR., 2 reads; Seq_All, 1 read per million (RPM); RT–PCR, according to the manufacturer’s instructions. Tests were considered uninterpretable when signal above background was observed in healthy pooled control samples assayed in parallel with patient samples. Heat maps indicate background-subtracted fluorescence after 1 h for most crRNAs (3 h time point is shown for HIV and HCV crRNAs). **c**, Heat map showing the full set of crRNAs designed to capture influenza N- sequence diversity. We tested 35 synthetic targets (10^4^ copies per μl) using 35 crRNAs. Grey, below detection threshold; green, fluorescence counts above threshold; orange outlines, subtypes; lowest row displays which targets are detected. Time, 3 h.

To test CARMEN in a more challenging context, we evaluated the HV panel against 58 plasma, serum, and throat and nasal swab samples from patients with a variety of confirmed infections. Each clinical sample was treated as an unknown and amplified using all 15 primer pools (Fig. 2e, Extended Data Fig. 7a). To increase testing throughput, PCR products were subsequently pooled in sets of three (five ‘metapools’ per patient sample) and tested with crRNAs from the HV panel (Extended Data Fig. 8a). As a gold-standard comparative readout, next-generation sequencing (NGS) was performed with more than 2 million reads per sample; of the 11,268 tests that were interpretable by both methods, 11,236 (99.7%) were concordant (Fig. 2f). We found that CARMEN identified the known infection in the majority of samples where NGS detected any sequences from these viruses, including complete concordance between CARMEN and NGS for dengue and Zika tests (Fig. 2g). CARMEN and NGS can also be compared on the basis of their ability to detect the sequence targeted by the CARMEN crRNA, revealing that CARMEN is more sensitive than NGS on a per-locus basis among the crRNA targets tested (Fig. 2h, Extended Data Fig. 8b). CARMEN’s overall sensitivity of detection, especially for diverse viruses, can be increased by the addition of crRNAs to cover additional loci and/or loci with sequence diversity, as we demonstrate with influenza A subtyping (Fig. 3). Notably, sequence heterogeneity at the target locus is a challenge that all targeted nucleic acid detection methods face, and CARMEN can overcome this through crRNA multiplexing. Finally, during our testing of samples from patients, both CARMEN and NGS identified specific viruses that were not previously known to exist in the samples (Fig. 2i, Extended Data Figure 8b). Thus, while it is clear the HV panel can be applied for surveillance of many viruses in parallel, it is important to recognize that integrating results from the HV panel with patient symptoms and medical expertise will be critical for the effective use of CARMEN testing in clinical settings.

Capitalizing on the specificity of Cas13 detection, we used CARMEN–Cas13 to discriminate all epidemiologically relevant serotypes of influenza A in parallel. Diversity within a viral species such as influenza A poses a substantial challenge to detection; an assay must correctly identify many distinct sequences within a group of strains, while remaining selective for that group. To discriminate the haemagglutinin (H) and neuraminidase (N) subtypes H1–H16 and N1–N9 of influenza A virus, we designed H and N amplicons that were sufficiently conserved to amplify with two parallel primer sets and used ADAPT to design specific sets of crRNAs to identify subtypes (Fig. 3a, see Methods for details). We tested the optimal crRNA from each set using synthetic consensus sequences from H1–H16 and N1–N9, and successfully identified these subtypes (Fig. 3b, c). We further tested our N-subtyping assay using synthetic sequences that collectively cover more than 90% of the sequence diversity within subtypes N1–N9, and identified 32 out of 35 (91.4%) of these sequences (Extended Data Fig. 8c). Finally, we validated our subtyping assay using 20 throat and nasal swabs from humans infected during the 2018–2019 flu season and were able to successfully subtype all of these infections, showing 100% concordance with results of reverse transcription with quantitative PCR performed by the Centers for Disease Control and Prevention and NGS performed in our laboratory (Fig. 3d, Methods, ‘Cas13-detection reactions’ under ‘General procedures’). On the basis of these results, our assay could potentially identify each of the 144 possible combinations of H1–H16 and N1–N9 subtypes.

**Fig. 4:**
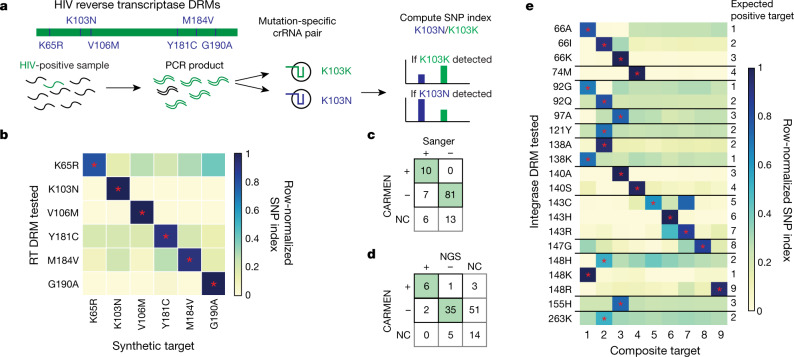
Multiplexed DRM identification with CARMEN–Cas13. **a**, Schematic for identification of DRMs in HIV reverse transcriptase using CARMEN–Cas13. **b**, Identification of six reverse transcriptase (RT) mutations using CARMEN–Cas13. **c**, **d**, Concordance between CARMEN–Cas13 and Sanger sequencing (**c**) and CARMEN–Cas13 and NGS (**d**), for identification of DRMs in patient plasma or serum samples for sequences with three or fewer mismatches relative to our design. NC, negative control. **e**, Identification of 21 integrase DRMs using CARMEN–Cas13. Heat maps indicate SNP indexes after 0.5–3 h of Cas13 detection, normalized by row. In **b**, **e**, synthetic targets were used at 10^4^ copies per μl. Asterisks indicate the synthetic target with the mutation.

**Extended Data Fig. 9:**
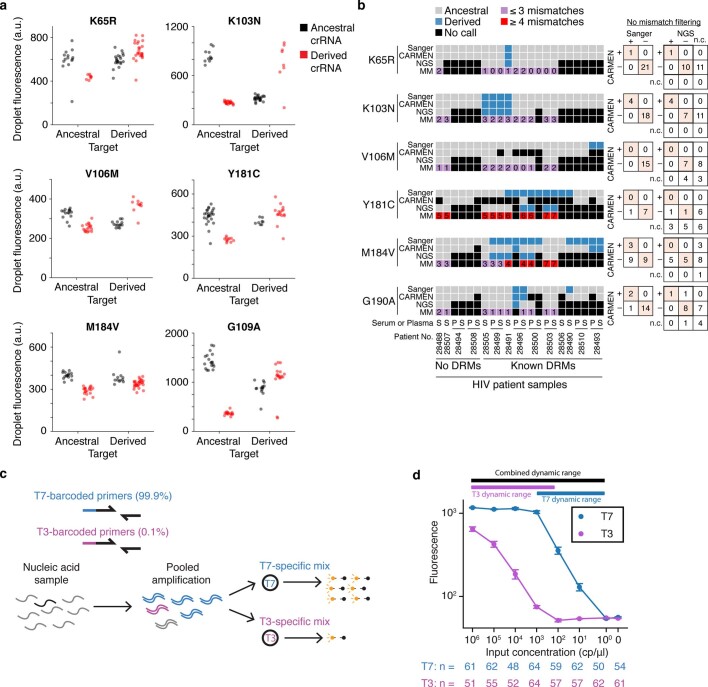
HIV reverse transcriptase mutation detection and future directions for CARMEN–Cas13. **a**, Distributions of droplet fluorescence for each HIV reverse transcriptase crRNA–target pair after 30 min in most cases; 3 h time point for V106M and M184V. SNP indices in Fig. 4b are calculated from the medians of these distributions. **b**, Comparison of prior expectation based on Sanger sequencing from the patient sample provider (Sanger), CARMEN testing (CARMEN), and NGS of RNA from each sample (NGS) for 22 patient samples infected with wild-type HIV (No DRMs) or HIV bearing known drug resistance mutations (known DRMs). In some cases, NGS revealed a high number of mismatches (MM) between the HIV sequence in the sample and the crRNA sequence used in the CARMEN HIV reverse transcriptase DRM panel. Summary tables at the right quantify concordance between CARMEN and Sanger sequencing or CARMEN and NGS. **c**, Quantitative CARMEN–Cas13 schematic showing amplification primers containing T7 or T3 promoters, leading to increased signal for the majority (T7) product after Cas13 detection. **d**, Increased dynamic range of detection using quantitative CARMEN–Cas13. Dynamic range is indicated using coloured bars above the graph. Error bars indicate s.e.m. Replicates (*n*) for T7 and T3 data are noted in colour-coded text beneath the plot.

The exquisite specificity of Cas13 also enables CARMEN–Cas13 to identify clinically relevant viral mutations in multiplex, such as those that confer drug resistance. To demonstrate this, we designed primer pairs tiling the HIV reverse transcriptase coding sequence and a set of crRNAs to identify six drug-resistance mutations (DRMs; Fig. 4a, Supplementary Table 2) that are prevalent in antiviral-naive patient populations^28^. Testing our designs against synthetic targets, we identified all six mutations in parallel (Fig. 4b, Extended Data Fig. 9a). We validated our reverse transcriptase-DRM assay on 22 samples from patients with HIV, some of which contained multiple mutations (Extended Data Fig. 9b), and demonstrated 90% concordance with Sanger sequencing results from the sample provider and 86% concordance with NGS we performed in parallel with CARMEN testing. In some cases, NGS revealed differences between our primer and crRNA designs and patient sequences, as we designed our assay against HIV subtype B, but tested it using samples obtained later from patients infected with HIV subtype G. Filtering by sequences with up to three mismatches relative to our design increased the concordance between CARMEN and Sanger sequencing (93%) and the concordance between CARMEN and NGS (93%) (Fig. 4c, d). To demonstrate the generalizability of our approach, we developed a CARMEN panel to test for 21 clinically relevant DRMs for HIV integrase^29^, the target of front-line HIV therapy, and identified all of these mutations in a set of 9 composite synthetic targets (Fig. 4e, Supplementary Table 2).

We have demonstrated a broad set of uses for CARMEN–Cas13 in differentiating viral sequences at the species, strain, and single-nucleotide polymorphism (SNP) levels and the capability to rapidly develop and validate highly multiplexed detection panels. More generally, CARMEN–Cas13 augments CRISPR-based nucleic acid-detection technologies by increasing throughput, decreasing reagent and sample consumption per test, and enabling detection over a wider dynamic range (Extended Data Fig. 9c, d). The flexibility and high throughput of CARMEN can accommodate the addition and rapid optimization of new amplification primers or crRNAs to existing CARMEN assays to facilitate detection of newly discovered pathogen sequences, as we demonstrated for SARS-CoV-2. Additionally, in the broader context of pathogen detection, discovery and evolution, CARMEN and NGS complement each other. CARMEN can rapidly identify infected samples for further sequencing to track the ongoing evolution of the virus, and newly identified sequences can inform the design of improved CRISPR-based diagnostics. In future, we imagine region- and outbreak-specific detection panels deployed to test thousands of samples from selected populations, including animal vectors, animal reservoirs, or patients presenting with symptoms. The adoption of such panels in connection with clinical care will require careful contextualized interpretation of results by experts. CARMEN enables CRISPR-based diagnostics at scale, a critical step toward routine, comprehensive disease surveillance to improve patient care and public health.

## Methods

No statistical methods were used to predetermine sample size. The experiments were not randomized. The investigators were not blinded to allocation during experiments and outcome assessment.

### Ethics statement

Human samples from patients with dengue, HCV, HIV and Zika were obtained commercially from Boca Biolistics under their ethical approvals. Influenza samples were obtained from the Centers for Disease Control and Prevention under their ethical approvals. All protocols subsequently performed by the researchers were approved as a Not Human Subjects Research determination no. NHSR-4318 issued by the Broad Institute of MIT and Harvard.

### General procedures

#### Synthetic targets

Synthetic DNA targets were ordered from Integrated DNA Technologies and resuspended in nuclease-free water. Resuspended DNA was serially diluted to 10^4^ copies per μl and used as inputs to PCR or RPA reactions.

#### CARMEN sample preparation

For all clinical samples and healthy human plasma, serum, urine, and nasal fluid, RNA was extracted from 140 μl of input material using the QIAamp Viral RNA Mini Kit (QIAGEN) with carrier RNA according to the manufacturer’s instructions. Samples were eluted in 60 μl of nuclease free water and stored at −80 °C until use. Ten microlitres of extracted RNA was converted into single-stranded cDNA in a 40-μl reaction. First, random hexamer primers were annealed to sample RNA at 70 °C for 7 min, followed by reverse transcription using SuperScript IV (Invitrogen) with random hexamer primers for 20 min at 55 °C. cDNA was stored at −20 °C until use. DNase treatment was not performed at any point during sample preparation.

#### Sequencing library preparation

Extracted viral nucleic acids were prepared for sequencing using library construction methods that have been previously described^30^, with a few differences noted below. Following extraction, double-stranded complementary DNA (cDNA) was created using random primers and SuperScript IV (Thermo Fisher Scientific) for first-strand synthesis and *Escherichia coli* polymerase I (NEB) for second-strand synthesis. Sequencing libraries were generated using the Nextera XT DNA Library Prep Kit (Illumina) with 10–16 cycles of PCR to introduce unique dual-index pairs. Libraries were then quantified using the KAPA Universal Complete Kit (Roche) and 12–18 samples were pooled for sequencing, including a no-input negative control. Samples were sequenced to >0.82 million read-mates using 2 × 75-bp paired-end reads from the Illumina NextSeq Reagent Kit v.2.5.

#### crRNA preparation

For viral detection (Figs 1–3), crRNAs were synthesized by Synthego and resuspended in nuclease-free water. For SNP detection (Fig. 4), crRNA DNA templates were annealed to a T7 promoter oligonucleotide at a final concentration of 10 μM in 1× Taq reaction buffer (New England Biolabs). This procedure involved 5 min of initial denaturation at 95 °C, followed by an anneal at 5 °C per minute down to 4 °C. SNP-detection crRNAs were transcribed from annealed DNA templates in vitro using the HiScribe T7 High Yield RNA Synthesis Kit (New England Biolabs). Transcriptions were performed according to the manufacturer’s instructions for short RNA transcripts, with the volume scaled to 30 μl. Reactions were incubated for 18 h or overnight at 37 °C. Transcripts were purified using RNAClean XP beads (Beckman Coulter) with a 2× ratio of beads to reaction volume and an additional supplementation of 1.8× isopropanol and resuspended in nuclease-free water. In vitro transcribed RNA products were then quantified using a NanoDrop One (Thermo Scientific) or on a Take3 plate with absorbance measured by a Cytation 5 (Biotek Instruments). Cas13a was recombinantly expressed and purified as described^7^ using Genscript, and was stored in storage buffer (600 mM NaCl, 50 mM Tris-HCl pH 7.5, 5% glycerol, 2mM DTT).

#### Nucleic acid amplification

Unless specified otherwise, amplification was performed by PCR using Q5 Hot Start polymerase (New England Biolabs) using primer pools (with 150 nM of each primer) in 20 μl reactions. Amplified samples were stored at −20 °C until use. For details about thermal cycling conditions, see ‘HV panel’, ‘Influenza A subtyping’ and ‘HIV DRMs’.

#### Cas13-detection reactions

For detection reactions, detection assays were performed with 45 nM purified *Leptotrichia wadei* Cas13a, 22.5 nM crRNA, 500 nM quenched fluorescent RNA reporter (RNAse Alert v2, Thermo Scientific), 2 μl murine RNase inhibitor (New England Biolabs) in nuclease assay buffer (40 mM Tris-HCl, 60 mM NaCl, pH 7.3) with 1 mM ATP, 1 mM GTP, 1 mM UTP, 1 mM CTP and 0.6 μl T7 polymerase mix (Lucigen). Input of amplified nucleic acid varied by assay with details as described in ‘Zika detection’, ‘HV panel’, ‘Influenza A subtyping’ and ‘HIV DRMs’. Detection mixes were prepared as 2.2× master mix, such that each droplet contained a 2× master mix after colour coding and a 1× master mix after droplet merging.

#### Colour coding, emulsification, and droplet pooling

For colour coding, unless specified otherwise, amplified samples were diluted 1:10 into nuclease-free water supplemented with 13.2 mM MgCl_2_ prior to colour coding to achieve a final concentration of 6 mM after droplet merging. Detection mixes were not diluted. Colour code stocks (2 µl) were arrayed in 96W plates (for detailed information on construction of colour codes, see ‘Colour code design, construction and characterization’.). Each amplified sample or detection mix (18 µl) was added to a distinct colour code and mixed by pipetting.

For emulsification, the colour-coded reagents (20 µl) and 2% 008-fluorosurfactant (RAN Biotechnologies) in fluorous oil (3M 7500, 70 µl) were added to a droplet generator cartridge (Bio Rad), and reagents were emulsified into droplets using a Bio Rad QX200 droplet generator or a custom aluminum pressure manifold.

For droplet pooling, a total droplet pool volume of 150 µl of droplets was used to load each standard chip; a total of 800 µl of droplets was used to load each mChip. To maximize the probability of forming productive droplet pairings (amplified sample droplet + detection reagent droplet), half the total droplet pool volume was devoted to target droplets and half to detection reagent droplets. For pooling, individual droplet mixes were arrayed in 96W plates. A multichannel pipette was used to transfer the requisite volumes of each droplet type into a single row of eight droplet pools, which were further combined to make a single droplet pool. The final droplet pool was pipetted up and down gently to fully randomize the arrangement of the droplets in the pool. The pooling step is rapid (<10 min), and small molecule exchange between droplets during this period does not substantially alter the colour codes (see Supplementary Discussion).

#### Loading, imaging and merging microwell arrays

Loading of standard chips was performed as described previously^31^. In brief, each chip was placed into an acrylic chip loader, such that the chip was suspended ~300–500 µm above the hydrophobic glass surface, creating a flow space between the chip and the glass. The flow space was filled with fluorous oil (3M, 7500) until loading; immediately before loading, fluorous oil was drained from the flow space. In a single pipetting step, the droplet pool was added to the flow space (Extended Data Fig. 2, step 3). The loader was tilted to move the droplet pool within the flow space until the microwells were filled with droplets. Fresh fluorous oil (3M 7500) without surfactant was used to wash the flow space (3 × 1 ml), the flow space was filled with oil, and the chip was sealed against the glass by screwing the loader shut (Extended Data Fig. 2, step 4). Additional oil (1 ml) was added to the loading slot, and the slot was sealed with clear tape (Scotch) to prevent evaporation.

For mChips, the back of an mChip was pressed against the lid of the mChip loader to adhere the chip to the lid and leave the microwell array facing out (Extended Data Fig. 5d, middle illustration). The lid was placed on the loader base, such that opposing magnets in the lid and base held the lid and chip suspended above the base (Extended Data Fig. 5d (right), f). Wingnuts on screws were used to push the lid toward the base until the flow space between the surface of the chip and base was ~300–500 µm (Extended Data Fig. 5d, right). The flow space was filled with fluorous oil (3M, 7500) until loading; immediately before loading, fluorous oil was drained from the flow space. In a single pipetting step, the droplet pool was added to the flow space by pipetting along the edge of the chip (Extended Data Fig. 5f, step 3). The loader was tilted to move the droplet pool within the flow space until the microwells were filled with droplets. Fresh fluorous oil (3M 7500) without surfactant was used to wash the flow space (3 × 1 ml). Two pieces of PCR film (MicroAmp, Applied Biosystems) were joined by placing the sticky side of one piece a few millimetres over the edge of the other piece. The sheet of PCR film was wetted with fluorous oil and set aside. Returning to the loader: the wingnuts were removed so the lid of the loader (with the mChip attached) could be removed from the base. The mChip was sealed against the sheet of wet PCR film in a single smooth motion (Extended Data Fig. 5f, step 4). The excess PCR film hanging over the edges of the chip was trimmed with a razor blade.

After chip loading, the colour code of each droplet was identified by fluorescence microscopy (Extended Data Figs. 2 (step 4), 5g). After imaging, the droplet pairs in each microwell were merged by passing the tip of a corona treater (Model BD-20, Electro-Technic Products) over the glass or PCR film (Extended Data Fig. 2, step 5). The merged droplets were immediately imaged by fluorescence microscopy (Extended Data Fig. 2, step 6) and placed in an incubator (37 °C) until subsequent imaging time points. All imaging was conducted on a Nikon TI2 microscope equipped with an automated stage (Ludl Electronics, Bio Precision 3 LM), LED light source (Lumencor, Sola), and camera (Hamamatsu, Orca Flash4.0, C11440, sCMOS). Unless otherwise noted, standard chips were imaged using a 2× objective (Nikon, MRD00025), while a 1× objective (Nikon, MRL00012) was used for mChips in order to reduce imaging time. The following filter cubes were used for imaging: Alexa Fluor 405: Semrock LED-DAPI-A-000; Alexa Fluor 555: Semrock SpGold-B; Alexa Fluor 594: Semrock 3FF03-575/25-25 + FF01-615/24-25; and Alexa Fluor 647: Semrock LF635-B. During imaging, the microscope condenser was tilted back to reduce background fluorescence in the 488 channel. Additionally, during experiments involving UV channel imaging, black cloth was draped over the microscope to reduce background signal from light scattered off the ceiling.

### Data analysis

#### General data analysis

Imaging data were analysed with custom Python scripts. Analysis consisted of three parts: (1) pre-merge image analysis to determine the identity of the contents of each droplet based on droplet colour codes; (2) post-merge image analysis to determine the fluorescence output of each droplet pair and map those fluorescence values back to the contents of the microwell; (3) statistical analysis of the data obtained in parts 1 and 2.

#### Pre-merge image analysis

The contents of each droplet were determined from images taken before droplet merging: a background image was subtracted from each droplet image, and fluorescence channel intensities were scaled so the intensity range of each channel was approximately the same. Droplets were identified using a Hough transform, and the fluorescence intensity of each channel at each droplet position was determined from a locally convolved image. Compensation for cross-channel optical bleed was applied, and all fluorescence intensities were normalized to the sum of the compensated 647 nm, 594 nm and 555 nm channels. For 4-channel datasets, analysis of 3-colour space was performed directly on normalized intensities. For 5-channel datasets, droplets were divided into UV intensity bins for downstream analysis (Extended Data Fig. 4). The 3-colour space within each UV bin was analysed separately. The 3-colour intensity vectors for each droplet were projected onto the unit 2-simplex, and density-based spatial clustering of applications with noise (DBSCAN) was used to assign labels to each colour code cluster. Manual clustering adjustments were made when necessary. For 5-channel datasets, UV intensity bins were recombined after assignments to create the full dataset.

#### Post-merge image analysis

Background subtraction, intensity scaling, compensation, and normalization were performed as in pre-merge analysis. Following image registration of pre- and post-merge images, the fluorescence intensity of the reporter channel at each droplet pair position was determined from a locally convolved image. The physical mapping of the fluorescent reporter channel onto the previously determined positions of each colour code served to assign the fluorescence signal in the reporter channel to the contents of each well. Quality filtering for appropriate post-merge droplet size (which excludes unmerged droplet pairs) and closeness of a droplet’s colour code to its designated colour code cluster (see Extended Data Fig. 4) was applied.

#### Statistical analysis

Heat maps were generated from the median fluorescence value of each crRNA–target pair. The performance of each guide was assessed by calculating a receiver operating characteristic (ROC) curve for the fluorescence distributions from on-target and all off-target droplets and determining the AUC.

#### SNP index calculation

The SNP index was calculated for each sample and each mutation by taking the ratio of the derived-allele-targeting crRNA and the ancestral-allele-targeting crRNA. In the heat maps, SNP indexes were normalized by row (in Fig. 4b, d).

#### Sequencing data analysis

Reads aligning specifically to the human genome were filtered using KrakenUniq 0.5.8, then deduplicated using clumpify.sh 38.61. Remaining reads were aligned to a KrakenUniq database (database, gs://sabeti-public-dbs/krakenuniq/krakenuniq.full.20190626.tar.zst; library, gs://sabeti-public-dbs/krakenuniq/krakenuniq.full.library.20190626.tar.zst). The output of this was used to compute the number of reads per million (rpm), and ≥1 rpm was considered a positive result.

For viral genome assembly, reads were demultiplexed and analysed using viral-ngs, which can be accessed at https://github.com/broadinstitute/viral-ngs/releases/tag/v1.25.0 (https://zenodo.org/record/3509008).

HIV genome assemblies were scaffolded against GenBank accession AF063224.1, which was also used as the reference for aligning all HIV reads for those samples with or without full genome assemblies. Thirteen HIV samples had the sufficiently high read depth (≥2 unique reads) to make consensus base calls at one or more of the regions targeted by the SNP assays. Consensus base calls in these regions were used to confirm the presence or absence of the SNP and determine the number of mismatches between each sample’s consensus HIV sequence and the crRNA. Each crRNA was aligned to each sample’s consensus sequence, and the number of mismatches was calculated excluding the synthetic mismatch, SNP-induced mismatch, or any mismatches that were G–U wobble base pairs from the total number. The ‘align_and_plot_coverage’ function in viral-ngs (wrapping BWA-MEM^32^, with options ‘--excludeDuplicates --minScoreToFilter 60’) was used to align human-depleted reads to AF063224.1; mean depth across each SNP amplicon for each sample was calculated, excluding zero values, and then was normalized to total raw reads per million of the sample.

### Zika detection

#### Nucleic acid amplification

Sample preparation was performed according to the method outlined in ‘CARMEN sample preparation’. For Zika virus detection (Fig. 1c, Extended Data Fig. 3b–e), RPA was used. RPA reactions were performed using the Twist-Dx RT–RPA kit according to the manufacturer’s instructions. Primer concentrations were 480 nM and MgAc_2_ concentration was 17 mM. For amplification reactions involving RNA, Murine RNase inhibitor (New England Biolabs) was used at a final concentration of 2 units per μl. All RPA reactions were incubated at 41 °C for 20 min unless otherwise stated. RPA primer sequences are listed in the supplementary data. RPA reactions were diluted 1:10 in nuclease-free water prior to colour coding.

#### Cas13-detection reactions

For Zika detection experiments (Fig. 1c), detection mixes were supplemented with MgCl_2_ at a final concentration of 6 mM prior to droplet merging. For comparison between CARMEN and SHERLOCK (Extended Data Fig. 3b, c), a Biotek Cytation 5 plate reader was used for measuring fluorescence of the detection reaction. Fluorescence kinetics were monitored using a monochromator with excitation at 485 nm and emission at 520 nm with a reading every 5 min for up to 3 h.

#### Colour coding, emulsification, loading, imaging, and merging microwell arrays

Amplified samples and detection mixes (18 μl) were colour coded using a subset of the 64-colour-code set. Colour coded solutions were emulsified into droplets, pooled, and loaded onto a standard chip (see ‘Colour coding, emulsification, and droplet pooling ‘ and ‘Loading, imaging and merging microwell arrays’ under ‘General procedures’). The chip was imaged with a 4× objective (Nikon, MRH00041) to identify colour codes, droplet pairs were merged, and reporter fluorescence in each well was measured by fluorescence imaging at 3 h. In this prototyping experiment, images were analysed without background subtraction.

#### Analysis of Zika detection

Bootstrapping was performed to estimate the number of crRNA–target pair replicates needed to reliably make a call. Sampling was done on two distributions: (1) crRNA-Target pairs expected to give a positive signal; (2) crRNA-control pairs expected to give a negative signal. A correct call was defined as the median of bootstrap samples from the positive distribution greater than the median of bootstrap samples in the negative distribution. One thousand bootstrap tests were performed for each sample size in the range of 1–15 samples. The fraction of correct calls was plotted as a function of bootstrap sample size.

### HV panel

#### Nucleic acid amplification

Sample preparation was performed according to the method outlined in CARMEN sample preparation. For the HV panel, amplification was performed using Q5 Hot Start polymerase (New England Biolabs) using primer pools (with 150 nM of each primer) in 20 μl reactions (see ‘HV panel design’ and Supplementary Data 2 for a detailed description of primer pool design and construction). Each target ultramer was amplified with the primer pool containing its corresponding primer pair(s). The following thermal cycling conditions were used: (1) initial denaturation at 98 °C for 2 min; (2) 45 cycles of 98 °C for 15 s, 50 °C for 30 s, and 72 °C for 30 s; (3) final extension at 72 °C for 2 min. For synthetic targets, each target was amplified with its corresponding primer pool. For clinical samples, each sample was amplified with all pools. For clinical samples, amplification reactions were diluted and mixed into five metapools as follows: pools 1–3, pools 4–6, pools 7–9, pools 10–12 and pools 13–15.

#### Cas13-detection reactions

Detection reactions were prepared as described in ‘Cas13-detection reactions’ under ‘General procedures’. In the first round of testing, all 169 crRNAs were used. In the second round, two high-performing crRNAs were omitted with no discernable negative effects on panel performance. For clinical samples, all 169 crRNAs were used, along with the HCV2 crRNA.

#### Colour coding, emulsification, loading, imaging and merging microwell arrays

Amplified samples and detection mixes (18 μl) were colour coded using a subset of the 1,050 colour code set. Colour coded solutions were emulsified into droplets, pooled, and loaded onto an mChip (see ‘Colour coding, emulsification and droplet pooling’ and ‘Loading, imaging and merging microwell arrays’ under ‘General procedures’). The chip was imaged with a 1× objective to identify colour codes, droplet pairs were merged, and reporter fluorescence in each well was measured by fluorescence imaging at 1 h and 3 h (see ‘Loading, imaging and merging microwell arrays’ under ‘General procedures’). Data were analysed as described in ‘Data analysis’.

For the full panel testing (169 × 169), a single replicate of the equivalent experiment conducted in 96W plates would require ~300 plates and >1 l of detection mix.

#### Threshold analysis of HV panel synthetic targets

For each crRNA, a threshold for detection was set at 3× s.d. above the background fluorescence. Cross-reactivity was defined as off-target reactivity above threshold. Low-reactivity was defined as no reactivity above threshold. Selective was defined as on-target reactivity above threshold and no cross-reactivity.

#### Analysis of patient sample testing with the 169-plex HV panel

To determine whether any crRNA in an experiment was uninterpretable due to signal above background in healthy control samples, the median signal across all crRNAs was calculated for each control sample. (Reactivity of the control samples across the 169-plex panel is expected to be very sparse, so the median value is a reliable measure of background signal.) Next, for each crRNA, a ratio was calculated of (numerator) the signal from the control sample with that crRNA and (denominator) the median for that control sample across all crRNAs. If any crRNA showed reactivity with a control sample that was >6x the median signal for that control sample, the crRNA was considered to be uninterpretable for that experiment. For each interpretable crRNA, the signal from each sample was divided by the median signal from the healthy control samples for that crRNA. Signal that was 6× above the median background signal was considered a positive result.

#### Commercial RT–PCR testing

RT–PCR testing for HCV and HIV was performed using the HCV TaqMan RT–PCR Kit and the HIV TaqMan RT–PCR Kit (both from Norgen Biosciences) according to the manufacturer’s recommendations (with 5 μl of RNA as input). RT–PCR testing for Zika and dengue was performed using the RealStar Dengue RT–PCR 3.0 kit and the RealStar Zika Virus RT–PCR Kit (both kits were RUO versions, from Altona Diagnostics), according to the manufacturer’s recommendations (with 10 μl of RNA as input). RT–PCR was performed using the Lyra Influenza A+B kit (Quidel) according to the manufacturer’s instructions (with 2.5 μl of RNA as input).

### Influenza A subtyping

#### Nucleic acid amplification

Sample preparation was performed according to the method outlined in ‘CARMEN sample preparation’. For the Influenza subtyping panel, amplification was performed using Q5 Hot Start polymerase (New England Biolabs) using primer pools (with 150 nM of each primer) in 20 μl reactions. The following thermal cycling conditions were used: (1) initial denaturation at 98 °C for 2 min; (2) 40 cycles of 98 °C for 15 s, 52 °C for 30 s, and 72 °C for 30 s; (3) final extension at 72 °C for 2 min. For the experiments shown in Fig. 3d, H and N amplification reactions were diluted together. H reactions were diluted 1:10 and N reactions were diluted 1:5 into nuclease-free water supplemented with 13.2 mM MgCl_2_ prior to colour coding. Detection reactions were prepared as described ‘Cas13-detection reactions’ under ‘General procedures’.

#### Colour coding, emulsification, loading, imaging, and merging microwell arrays

Amplified samples and detection mixes (18 μl) were colour coded using a subset of the 64-colour-code set. Colour-coded solutions were emulsified into droplets, pooled, and loaded onto a standard chip (see ‘Colour coding, emulsification and droplet pooling’ and ‘Loading, imaging and merging microwell arrays’ under ‘General procedures’). The chip was imaged with a 2× objective to identify colour codes, droplet pairs were merged, and reporter fluorescence in each well was measured by fluorescence imaging at 1 or 3 h (see ‘Loading, imaging and merging microwell arrays’ under ‘General procedures’). Data were analysed as described in ‘Data analysis’.

#### Analysis of patient sample testing with the influenza-subtyping panel

The threshold for each crRNA may be set individually, as the reactivity of a crRNA is sequence-specific. For H-subtyping crRNA, the signal from each sample was divided by the median signal from the healthy control samples for that crRNA. Signal that was 6× above the median background signal was considered a positive result. The N-subtyping crRNAs are less reactive, so a more sensitive threshold is necessary to accurately differentiate signal from background. For each N-subtyping crRNA, the median and standard deviation of the control samples was calculated, and a threshold of 7× s.d. above the median was used to determine signal above background.

### HIV DRMs

#### Nucleic acid amplification

Sample preparation was performed according to the method outlined in ‘CARMEN sample preparation’. For the HIV DRM panels, amplification was performed using Q5 Hot Start polymerase (New England Biolabs) using primer pools (with 150 nM of each primer) in 20 μl reactions. The following thermal cycling conditions were used: (1) initial denaturation at 98 °C for 2 min; (2) 40 cycles of 98 °C for 15 s, 52 °C for 30 s, and 72 °C for 30 s; (3) final extension at 72 °C for 2 min. For the experiments shown in Fig. 4, even and odd reactions were diluted together at 1:10 into nuclease-free water supplemented with 13.2 mM MgCl_2_ prior to colour coding. Detection reactions were prepared as described in ‘Cas13 detection reactions’ under ‘General procedures’.

#### Colour coding, emulsification, loading, imaging and merging microwell arrays

Amplified samples and detection mixes (18 μl) were colour coded using a subset of the 64-colour-code set. Colour-coded solutions were emulsified into droplets, pooled and loaded onto a standard chip (see ‘Colour coding, emulsification and droplet pooling’ and ‘Loading, imaging and merging microwell arrays’ under ‘General procedures’). The chip was imaged with a 2× objective to identify colour codes, droplet pairs were merged, and reporter fluorescence in each well was measured by fluorescence imaging at 30 min or 3 h (see ‘Loading, imaging and merging microwell arrays’ under ‘General procedures’). Data were analysed as described in ‘Data analysis’.

#### Analysis of patient sample testing with the HIV RT DRM panel

In order for CARMEN to make a SNP call, the reactivity of one of the crRNAs (ancestral or derived) for that SNP must be above background. To filter out ‘no-call’ results, the sum of the ancestral and derived crRNAs for each SNP was divided by the sum of the minimum ancestral and minimum derived signal for those crRNAs. The no-call threshold was 1.2× the sum of minimum values. For tests where a call could be made, the background-subtracted derived signal was divided by the background-subtracted ancestral signal. A threshold for each SNP was set based on the ratios from ancestral and derived synthetic sequences run in parallel with the patient samples, and the thresholds ranged from 1–3.

### HV panel design

#### Overview

A schematic overview of the HV panel sequence design strategy is shown in Extended Data Fig. 5h. In brief, the design pipeline consisted of viral genomes segment alignment and PCR amplicon selection followed by crRNA design that accounts for cross-reactivity. Finally, PCR primers were pooled by genus. All sequences are in Supplementary Data 2.

#### Viral genome segment alignment

Viral genome neighbours were downloaded from NCBI. Each segment of each viral species was aligned using mafft v.7.31^33^ with the following parameters: --retree 1 --preservecase. Alignments were curated to remove sequences that were assigned the wrong species, reverse-complemented, or came from the wrong genome segment. The aligned genome segments can be found at the following link: https://storage.googleapis.com/sabeti-public/carmen_design/hav10_fft1_alignments.tar.gz.

#### PCR amplicon selection

Potential PCR binding sites were identified by using ADAPT with a window size of 20 nucleotides, and a coverage requirement of 90% of the sequences in the alignment (Metsky et al., manuscript in preparation). Potential pairs of primer binding sites within a distance of 70 to 200 nucleotides were selected. These sets of potential primer pairs were input into primer3 v.2.4.0^27^ to see if suitable PCR primers could be designed for amplification. Primer3 was run using the following parameters: PRIMER_TASK=generic, PRIMER_EXPLAIN_FLAG=1, PRIMER_MIN_SIZE=15, PRIMER_OPT_SIZE=18, PRIMER_MAX_SIZE=20, PRIMER_MIN_GC=30.0, PRIMER_MAX_GC=70.0, PRIMER_MAX_Ns_ACCEPTED=0, PRIMER_MIN_TM=52.0, PRIMER_OPT_TM=54.0, PRIMER_MAX_TM=56.0, PRIMER_MAX_DIFF_TM=1.5, PRIMER_MAX_HAIRPIN_TH=40.0, PRIMER_MAX_SELF_END_TH=40.0, PRIMER_MAX_SELF_ANY_TH=40.0, PRIMER_PRODUCT_SIZE_RANGE=70-200. A list of potential amplicons was generated by parsing the primer3 output file, filtering to ensure that the maximum difference in melting temperature between any pair of forward and reverse primers was less than 4 °C (so that all primers in the pool would have similar PCR efficiency). This list of potential amplicons was then scored based on the average pairwise penalty between all pairs of forward and reverse primers in the design, as measured by primer3. The amplicon with the highest score from each species was chosen for crRNA design (see Supplementary Data 2 for primer and amplicon sequences).

#### crRNA design

We used a software package called ADAPT (Metsky et al., manuscript in preparation), which implements an algorithm to design crRNAs, such that the number of them approximates the minimum number of crRNAs that bind to 90% of the sequences within a 40 nt window of each amplicon alignment, allowing for up to one mismatch between each crRNA and target sequence, and allowing for G–U pairing. These crRNA sets are designed in silico by the algorithm to avoid cross-reactivity at the family level, requiring 3 or more mismatches for >99% of sequences in the other species within the same family, allowing for G–U pairing. This stringent threshold was chosen to ensure high specificity for the HV assay. For closely related viral genuses (enterovirus, and poxvirus), the algorithm selected regions where the majority consensus sequence for each species differed and only considered crRNAs in windows where there was sufficient sequence divergence at the majority consensus level (see Supplementary Data 2 for crRNA sequences).

#### Primer pooling

We designed primers (as described above) for a set of 169 species that have at least one segment with ≥10 sequences in the downloaded data, hereafter referred to as the HV panel 10 version 1 or HV10-v1. Owing to limitations of multiplexed PCR, the 210 primer pairs that we designed for the 169 HV10 species in the version 1 design were split into 15 primer pools, described in more detail below.

#### Conserved primer pool

We selected 14 conserved species as a pilot experiment to test our primer design algorithm and pooling strategy. Species are listed in Supplementary Data File 2. These species were combined into a single conserved primer pool at 150 nM final concentration. This is pool 1, as shown in Fig. 2c.

#### Diverse primer pool

Of the 169 HV10 species, 164 have designs with 3 or fewer primer pairs (total of 187 primer sequences required to cover these 164 species: 145 have 1 primer pair, 15 have 2 primer pairs, and 4 have 3 primer pairs). There were four species that required more than three primer pairs: lymphocytic choriomeningitis virus (7 primer pairs), norovirus (4 primer pairs), betapapillomavirus 2 (6 primer pairs) and *Candiru phlebovirus* (6 primer pairs). These four species were combined into a single ‘diverse’ primer pool at 150 nM final concentration. This is pool 2, as shown in Fig. 2c.

#### Degenerate primer pool

For 167 of the 169 HV10 species, it was possible to design primer sets using ADAPT/primer3 that cover >90% of the genomes in the database with fewer than 10 primer pairs. However, for two species (simian immunodeficiency virus and Sapporo virus) it was not possible to identify sufficiently conserved pairs of primer binding sites using our computational design strategy. Instead, we designed primers with several degenerate bases to capture the extensive sequence diversity, and manually identified amplicons. These two primer pairs were used in a degenerate primer pool at 600 nM final concentration. This is pool 3, as shown in Fig. 2c.

#### Remaining primer pools

For the remaining 149 HV10 species, we pooled primers by genus, such that each pool contained species from 1–3 viral genuses (see Supplementary Data 2 for details). The primers for one species in pool 4 (Torque teno Leptonychotes weddellii virus-1) contain some degenerate bases, and were designed manually. These primers were used at 150 nM final concentration.

#### Coronavirus primer pool

Primers used in the coronavirus panel are indicated in Supplementary Data 2. These primers were used at 150 nM final concentration.

#### Version one design analysis

In the analysis of version one performance, it was discovered that crRNA 136 had inadvertently been designed against target 128. Both crRNA 128 and crRNA 136 selectively react with target 128, and were thus counted as selective crRNAs. To computationally analyse the expected version one design performance, spacer target sequences and primers were aligned using bwa 0.7.17-r1188^32^ against the majority consensus sequences of each of the 169 viral genomes. Alignments with insertions or deletions were not permitted. Primers and crRNAs activity were scored using the alignments output by bwa. The score for both primers and crRNAs was the number of matching bases between the crRNA and target sequence, except for crRNA activity the score also counted crRNA-target pairs of A-G and C-T to include G-U pairing. Score cut-offs were 17 for primers and 27 for crRNAs. This yielded a 169 × 169 predicted reactivity matrix for the primers, and another matrix for the crRNAs. This matrix was summed to calculate the expected number of targets that each primer or crRNA would react with. A score of 0 was categorized as low activity, a score of 1 as perfect activity, and a score >1 as cross-reactivity.

#### Version two redesign

After testing the HV10-v1 design, 3 amplicons were redesigned: orthohepesvirus A, rhinovirus A and rhinovirus B. The newly designed primers were re-pooled to create pools 8v2 and 12v2, and new crRNA sequences were designed to target these amplicons. On the basis of the results of the HV10-v1 testing, we redesigned crRNAs within the existing v1 amplicons for 14 species (see Supplementary Data 2 for details).

### Influenza A subtyping design

#### Primer design

N (neuraminidase) primers were based on the majority consensus sequence for each subtype (9 primer pairs) in a single pool. We used ADAPT to design H (hemagglutinin) primers covering at least 95% of the sequences within each subtype. In total, there were 45 primers (15 forward primers and 30 reverse primers) in a single pool. See Supplementary Data 2 for details.

#### crRNA design

Sets consisting of a small number (1–5) of crRNA sequences were designed to selectively target individual H or N subtypes using ADAPT (Metsky et al., manuscript in preparation). We improved our design approach throughout the process by incorporating new features into each round of design. In the first round of design, we only designed H crRNAs, and required that all crRNAs could hybridize with 90% of all sequences, allowing for up to 1 mismatch. crRNAs in a set could be positioned anywhere in the amplicon. In the second round of design, we designed crRNAs for both H and N and restricted the positions of crRNAs within a set (to within a 91-nt window for H and 35-nt window for N) as some positions within the amplicon were more conserved between subtypes than others. As in round 1, in round 2 we required that all crRNAs could hybridize with 90% of all sequences, allowing for up to 1 mismatch. In addition, we weighted the coverage of our designs towards more recent years by using an exponential decay parameter for sequences from before 2017. In the third round, we used a differential design approach in which all crRNAs were required to have at least 3 mismatches against at least 99% of sequences within any other subtype. In the fourth round, we accounted for G–U pairing in hybridization, and raised the target threshold to 95% of sequences in each subtype, allowing for up to 1 mismatch. Each round of designs was tested experimentally, and high-performing crRNAs between designs were used in combination. H required four rounds of design, while N only required two rounds (rounds two and three). Oligonucleotide sequences are listed in Supplementary Data 2.

### HIV DRM panel design

#### Primer design

We used a primer pooling strategy in which primer pairs were divided into overlapping odd and even primer pools on the basis of the locations of DRMs within the reverse transcriptase and integrase genes^34^. This allowed for all mutations to be contained in at least one amplicon, without creating any issues during amplification. Primer sequences were designed using primer3 v.2.4.0 with the following parameters: PRIMER_PRODUCT_OPT_SIZE=150, PRIMER_MAX_GC=70, PRIMER_MIN_GC=30, PRIMER_OPT_GC_PERCENT=50, PRIMER_MIN_TM=55, PRIMER_MAX_TM=60, PRIMER_DNA_CONC=150, PRIMER_OPT_SIZE=20, PRIMER_MIN_SIZE=16, PRIMER_MAX_SIZE=29. Amplicon lengths ranged between 150 and 250 nt. All primer sequences are in Supplementary Data 2.

#### crRNA design

Pairs of crRNAs were designed for HIV DRM identification using three different strategies: mutation in position 3 and synthetic mismatch in position 5, DRM codon in positions 3–5 and synthetic mismatch in position 6, and DRM codon in positions 4–6 with synthetic mismatch at position 3. Sequences were designed on the basis of the HIV subtype B consensus sequence, using the most-commonly used codons for each respective amino acid in the Stanford HIV Drug Resistance Database^35^. All designs were experimentally tested, and the best-performing design was chosen for the final panel.

### Microwell-array chip design and fabrication

#### Microwell-array design

Microwell dimensions were optimized by empirical testing to balance droplet loading speed (faster with larger wells) and droplet–droplet closeness inside a microwell (better merging with smaller wells). For droplets made from PCR amplification reactions or Cas13-detection mix, the optimal microwell geometry was achieved by joining two circles with diameters of 158 µm and an overlap of 10% (Extended Data Fig. 1c). The microwells were designed with a minimum distance of 37 µm between each well to facilitate consistent chip fabrication without PDMS tearing (see ‘Microwell chip fabrication’). Standard chips have a total microwell array that is 6.0 × 5.5 cm (51,496 microwells); the loading slot partially obscures the microwell array, reducing the functional array size to 6.0 × ~4.5 cm (~42,400 microwells) (Extended Data Fig. 1d). mChips have a microwell array that is 12 × 9.1 cm, bearing 177,840 microwells (Extended Data Fig. 5a). The mChip microwell array is surrounded by a 0.1–0.3 cm border of unpatterned PDMS to facilitate a robust seal around the edge of the chip. The total mChip dimensions were designed to maximize the number of wells that can be imaged on the area of a standard microscope stage (16 × 11 cm opening, Bio Precision LM Motorized Stage, Ludl Electronics), while still allowing the chip to be fabricated using standard silicon wafers (15 cm diameter) (Extended Data Fig. 5b).

#### Microwell chip fabrication

PDMS chips were fabricated according to standard hard- and soft-lithography practices using acrylic moulds to achieve consistent chip dimensions; the fabrication of standard size chips has been described previously^8^. For mChips, 150 mm wafers (WaferNet, no. S64801) were washed on a spin coater (Model WS-650MZ-23NPP, Laurell Technologies) at 2,500 rpm, once with acetone and once with isopropanol. Photoresist (SU-8 2050, MicroChem) was spin-coated onto each wafer in a two-step process: (1) 30 s, 500 rpm, acceleration 30; (2) 59 s, 1,285 rpm, acceleration 50. Wafers were baked at 65 °C for 5 min and, subsequently, at 95 °C for 18 min. After a 1-min cooling period, the coated wafer was placed under the appropriate photomask and irradiated (5 × 3 s, 350 W, Model 200, OAI). The wafer was baked again at 65 °C for 3 min and 95 °C for 9 min. After 1 min of cooling, the wafer was incubated for 5 min under SU-8 developer. The developer was removed by spinning at 2,500 rpm, and acetone and isopropanol washes were applied directly to the spinning wafer to remove excess developer and photoresist. Each wafer was characterized by visual inspection under a light microscope and profilometry to measure feature dimensions (Contour GT, Bruker). Wafers were placed inside acrylic moulds and secured with magnets (Extended Data Fig. 5b). To fabricate chips from the moulds, PDMS was mixed (Thinky planetary vacuum mixer, ARV-310) and poured into the mould, and the entire mould was placed under house vacuum for 3–5 min. The mould was closed with an acrylic lid to achieve uniform chip thickness, and the chips were baked for at least 2 h. After the chip was removed from the mould, the surface of the chip bearing the microwell array and the sides (but not the back of the chip opposite the microwell array) were coated with 1.5 µm Parylene C (Paratronix/MicroChem). Chips were stored in plastic bags at room temperature until use.

#### Acrylic device fabrication

Moulds^8^ and loaders^31^ for standard chip production and handling were constructed as described previously. Similar methods were used to construct moulds and loaders for mChip (Extended Data Fig. 5b, d). In brief, 12 inch × 12 inch cast acrylic sheets (¼ inch or 1/8 inch, clear or black) were purchased from Amazon (Small Parts, no. B004N1JLI4). Mould and loader designs were created in AutoCAD (AutoDesk), and parts were cut using an Epilog Fusion M2 laser cutter (60 W). Acrylic parts were fused together by wetting with dichloromethane (Sigma Aldrich). N42 Neodymium disc magnets (Applied Magnets) were added to devices with epoxy (Loctite, Metal/Concrete). Cap screws (M4 × 25), nuts (M4), and washers (M4) were purchased from Thorlabs.

### Colour code design, construction, and characterization

#### Colour code design

Colour codes served as optical unique solution identifiers for each reagent (e.g. detection mix or amplified sample) that was emulsified into droplets. The original 64-colour-code set was made from ratios of 3 fluorescent dyes, such that the total concentration of the three dyes ([dye 1] + [dye 2] + [dye 3]) was constant and served as an internal control to normalize for variation in illumination across the field of view or at different locations on the chip^8^. The working total dye concentration for the 64-colour-code set was 1–5 µM, as described previously^8^. The 1,050 colour codes were designed by (1) increasing the total working concentration of the 3 fluorescent dyes to 20 µM, such that 210 colour codes could be faithfully identified in 3-colour space (Extended Data Fig. 4a, b), and (2) adding a fourth fluorescent dye at one of 5 concentrations (0, 3, 7, 12 or 20 µM) to multiply the 210 codes by 5 (Extended Data Fig. 4a). In this design, each of the four dye intensities is normalized to the sum of the first three fluorescent dyes.

#### Colour code construction

The standard 64-colour-code set (50 µM stock concentration; 1–5 µM working concentration) was constructed as previously described^8^ (Supplementary Data 1). The 210 colour codes (400 µM stock concentration; 20 µM working concentration, see Supplementary Data 1 for ratios) were constructed using similar methods, as follows. Alexa Fluor 647 (AF647), Alexa Fluor 594 (AF594), Alexa Fluor 555 (AF555), and Alexa Fluor 405 NHS ester (AF405–NHS) (Thermo Fisher) were diluted to 25 mM in DMSO (Sigma). Since the molar masses of these dyes are proprietary, the following approximate masses provided by the manufacturer were used for calculations: AF647: 1,135 g mol^−1^; AF594: 1,026 g mol^−1^; AF555: 1,135 g mol^−1^; AF405–NHS: 1,028 g mol^−1^. Dye stocks in dimethyl sulfoxide (DMSO) were further diluted to 400 µM in DNase/RNase-free water (Life Technologies). Alexa Fluor 405 NHS ester was incubated at room temperature for 1 h to allow hydrolysis of the NHS ester and generate Alexa Fluor 405 (AF405). Custom MATLAB scripts were used to calculate the dye volumes to combine to evenly distribute 210 colour codes across the 3-colour space (Supplementary Data 1). Three-colour dye combinations (made from AF647, AF594 and AF555) were constructed in 96 well plates (Eppendorf) using a Janus Mini liquid handler (Perkin Elmer). To construct 1,050 colour codes, AF405 was manually diluted to five concentrations (0, 60, 140, 240 and 400 µM), and each concentration was arrayed across a 96 well plate. Each of the 210 colour codes (10 µl) and AF405 (10 µl) were combined and mixed in a fresh 96 well plate using a Bravo liquid handler (Agilent). The final stock concentration of the sum of AF647, AF594 and AF555 was 200 µM; the final concentrations of AF405 were 0, 30, 70, 120 and 200 µM. Stocks were diluted 1:10 into amplified samples or detection mixes for use.

#### Characterization of 1,050-colour-code set

Each colour code was diluted 1:10 in LB broth (a medium that yields droplets of similar size to droplets made from PCR products and detection reagents) to a final total 3-dye concentration of 20 µM. Each solution was emulsified into droplets as described in ‘Colour coding, emulsification and droplet pooling’ under ‘General procedures’. The 1,050-colour-code set was characterized in 3-colour space and along the 4th colour dimension as described below.

#### Characterization of the 1,050-colour-code set in three-colour space

The fidelity of the colour code strategy in three-colour space was measured as described previously^8^. Each colour code in three-colour space was assigned to one of three chips. Assignments were made to maximize the separation between the colour codes on any chip, and each chip received a third of the colour codes (70 total) (Extended Data Fig. 4b, c). Droplets from colour codes assigned to Chip 1 (70 3-colour codes × 5 UV concentrations = 350 droplet emulsions) were pooled (see ‘Colour coding, emulsification and droplet pooling’ under ‘General procedures’) and loaded onto a standard chip (see ‘Loading, imaging and merging microwell arrays’ under ‘General procedures’). Chips 2 and 3 were prepared in a similar manner. The chips were imaged (see ‘Loading, imaging and merging microwell arrays’ under ‘General procedures’; note that no merging was performed in colour code characterization experiments), and each droplet was computationally assigned to a colour code cluster. The experimental results from chips 1, 2 and 3 served as ‘ground truth’ assignments. The data from chips 1, 2 and 3 were then computationally combined, effectively increasing the density of colour code clusters in 3-colour space, and the droplets were reassigned to colour code clusters in this more crowded 3-colour space (Extended Data Fig. 4b, c). Finally, a sliding distance filter was applied to remove droplets at the edges of clusters or in between clusters, and the droplets were reassigned to colour code clusters (Extended Data Fig. 4b, f). The sliding distance filter refers to a radius around each cluster centroid that is used to remove droplets that fall in the space between clusters (Extended Data Fig. 4f). The radius may be larger (to include more droplets) or smaller (to more stringently filter out droplets). New assignments were compared to ground truth assignments to measure the percent of droplets that would be misclassified if the colour codes were not separated over three chips (Extended Data Fig. 4d, e). In the work presented here, the radius of the sliding distance filter was set to achieve at least 99.5% correct classification in the test dataset, corresponding to the removal of 6% of droplets.

#### Characterization of the 1,050-colour-code set along the fourth colour dimension

The five concentrations of the fourth fluorescent dye were divided between two chips (chip 1: 0, 7 and 20 µM; chip 2: 3 and 12 µM) (Extended Data Fig. 4g). Droplets from dye intensities assigned to chip 1 (3 UV intensities × 210 colour codes = 620 emulsions) were pooled (see ‘Colour coding, emulsification and droplet pooling’ under ‘General procedures’) and loaded onto a standard chip (see ‘Loading, imaging and merging microwell arrays’ under ‘General procedures’). Chip 2 was prepared in a similar manner but with fewer pooled emulsions (2 UV intensities × 210 colour codes = 420 emulsions). The chips were imaged (see ‘Colour coding, emulsification and droplet pooling’ under ‘General procedures’; note that no merging was performed in colour code characterization experiments), and each droplet was computationally assigned to a UV intensity bin. The experimental results from chips 1 and 2 served as ground truth assignments. The data from chips 1 and 2 were then computationally combined, effectively increasing the density of UV intensity bins along the 4th-colour dimension, and the droplets were reassigned to UV intensity bins in this more crowded space (Extended Data Fig. 4g). Finally, a sliding distance filter was applied to remove droplets at the edges of intensity bins or in between intensity bins, and the droplets were reassigned to UV intensity bins (Extended Data Fig. 4g). New assignments were compared to ground truth assignments to measure the percent of droplets that would be misclassified if the UV intensities were not separated over three chips (Extended Data Fig. 4g). As classification in the fourth colour dimension is sufficiently high (>99.5% accurate) without filtering, no filtering in the fourth colour dimension was applied to the experimental data.

### Reporting summary

Further information on research design is available in the Nature Research Reporting Summary linked to this paper.

## Online content

Any methods, additional references, Nature Research reporting summaries, source data, extended data, supplementary information, acknowledgements, peer review information; details of author contributions and competing interests; and statements of data and code availability are available at 10.1038/s41586-020-2279-8.

## Supplementary information

Supplementary Information This file contains Supplementary Tables 1 and 2, a Supplementary Discussion of CARMEN’s sensitivity and specificity, experimental design, microwell array statistics, fidelity of colour code analysis, cost and sample consumption analysis, CARMEN workflow time, reduction in liquid handling steps, and human associated virus (HV) panel performance.

Reporting Summary

Supplementary Data 1 Information for preparing colour codes.

Supplementary Data 2 A list of oligonucleotides used in this study.

Supplementary Data 3 Human associated virus panel synthetic target testing data.

Supplementary Data 4 A list of clinical samples used and associated metadata.

Supplementary Data 5 Human associated virus panel patient sample data.

Supplementary Data 6 Flu subtyping patient sample data.

Supplementary Data 7 HIV RT patient sample data.

Supplementary Data 8 Data on number of replicates per experiment

## Figures and Tables

**Extended Data Table 1:**
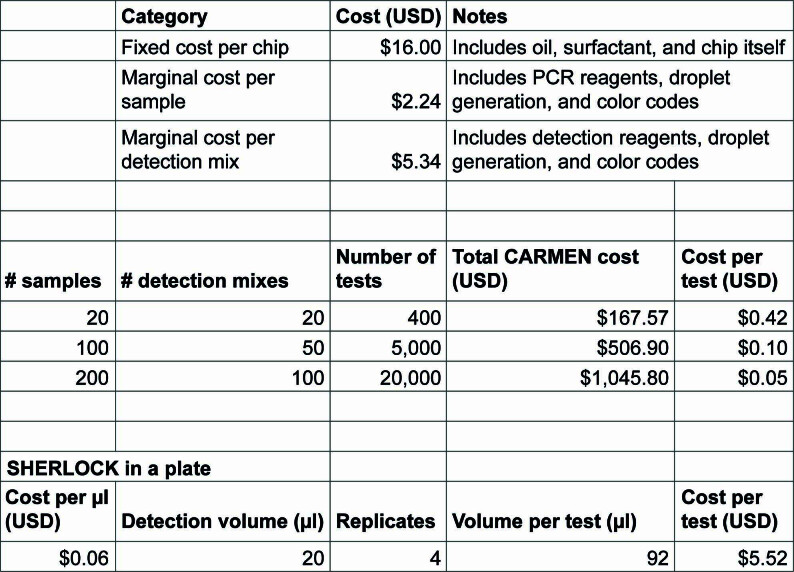
Consumables cost calculation concerning CARMEN–Cas13

## Data Availability

The CARMEN datasets generated during and/or analysed during the current study are available from the corresponding authors on reasonable request. Fluorescence values for rounds 1 and 2 of the HV panel testing and patient sample testing are included in Supplementary Data 3–7. Viral sequencing data have been deposited in the Sequence Read Archive under accession number PRJNA623215.

## Code availability

The code used for CARMEN data analysis is available on GitHub at https://github.com/blaineylab/kChip/tree/kchip_UV and https://github.com/blaineylab/kChip/tree/kchip_clustering.
