# Supplementary material for: Massively multiplexed nucleic acid detection with Cas13
Source: Nature. 2020 Apr 29;582(7811):277–82. doi: 10.1038/s41586-020-2279-8 (PMC7332423; doi:10.1038/s41586-020-2279-8)
Supplement: Supplementary file 1 — This file contains Supplementary Tables 1 and 2, a Supplementary Discussion of CARMEN’s sensitivity and specificity, experimental design, microwell array statistics, fidelity of colour code analysis, cost and sample consumption analysis, CARMEN workflow time, reduction in liquid handling steps, and human associated virus (HV) panel performance. [file 41586_2020_2279_MOESM1_ESM.pdf]

nature

https://doi.org/10.1038/s41586-020-2279-8

Supplementary information

Massively multiplexed nucleic acid detection with Cas13

In the format provided by the authors and unedited

Cheri M. Ackerman, Cameron Myhrvold✉, Sri Gowtham Thakku, Catherine A. Freije, Hayden C. Metsky, David K. Yang, Simon H. Ye, Chloe K. Boehm, Tinna-Sólveig F. Kosoko-Thoroddsen, Jared Kehe, Tien G. Nguyen, Amber Carter, Anthony Kulesa, John R. Barnes, Vivien G. Dugan, Deborah T. Hung, Paul C. Blainey✉ & Pardis C. Sabeti

Supplementary Discussion for

Massively multiplexed nucleic acid detection with Cas13

Cheri M. Ackerman*, Cameron Myhrvold*§, Sri Gowtham Thakku†, Catherine A. Freije†, Hayden C. Metsky, David K. Yang, Simon H. Ye, Chloe K. Boehm, Tinna-Sólveig F. Kosoko-Thoroddsen, Jared Kehe, Tien G. Nguyen, Amber Carter, Anthony Kulesa, John R. Barnes, Vivien G. Dugan, Deborah T. Hung, Paul C. Blainey‡§, and Pardis C. Sabeti‡

* equal contributions and listed alphabetically
† equal contributions
‡ equal contributions and listed alphabetically
§ to whom correspondence should be addressed: C.M.: cmyhrvol@broadinstitute.org, P.C.B: pblainey@broadinstitute.org

Contents

Supplementary Tables 1-2

Supplementary Table 1: Droplet pairing and filtering statistics for rounds 1 and 2 of human associated virus panel testing.
Supplementary Table 2. List of HIV drug-resistance mutations tested in this study.

Supplementary Discussion

1. **The sensitivity of CARMEN is equivalent to that of SHERLOCK**
2. **The specificity of CARMEN is derived from Cas13-crRNAs, not primers**
3. **Flexibility of experimental design**
4. **Microwell array statistics**
    - A. **Factors affecting the number of productive droplet pairs per chip**
    - B. **Factors affecting the number of replicates per test required to make an accurate call**
    - C. **How to calculate the number of tests that can be performed on one chip**
    - D. **False negatives and false positives due to color code misclassification**
5. **Fidelity of color code analysis**
    - A. **Controlling exchange of solutes between droplets during pooling**
    - B. **Color code analysis**
6. **Cost and sample consumption analysis**
    - A. **Reagent costs**
    - B. **Equipment costs**
    - C. **Labor costs**
    - D. **Cost scaling**
    - E. **Sample volume requirements**
7. **CARMEN workflow time**
8. **Reduction in liquid handling steps using CARMEN**
9. **Human associated virus panel performance**
    - A. **Selection of optimal crRNAs for testing**
    - B. **Cross-contamination**
    - C. **Sequence coverage**
    - D. **Testing of unknown samples**
    - E. **Comparing CARMEN and sequencing data**
    - F. **Detection of unexpected viruses with CARMEN**

References

		Droplet pairs	crRNA+Target pairs	Yield (%)	Filtered pairs	Passed filter (%)	crRNAs	Targets	Tests
**Testing round 1**	Chip1	154,451	74,518	48.2	67,773	90.9	22	200	4400
	Chip2	154,331	74,344	48.2	65,868	88.6	22	200	4400
	Chip3	156,621	75,657	48.3	69,308	91.6	23	200	4600
	Chip4	157,090	75,734	48.2	67,377	89.0	22	200	4400
	Chip5	151,248	72,694	48.1	68,311	94.0	19	190	3610
	Chip6	142,738	67,744	47.5	63,156	93.2	19	190	3610
	Chip7	141,292	67,143	47.5	63,048	93.9	19	190	3610
	Chip8	155,889	75,361	48.3	71,141	94.4	18	190	3420
**Total**		**1,213,660**	**583,195**		**535,982**				
**Average (per chip)**		**151,708**	**72,899**	**48.0**	**66,998**	**92.0**			
**Testing round 2**	Chip1	146,333	67,286	46.0	62,282	92.6	23	189	4347
	Chip2	151,635	71,971	47.5	67,212	93.4	24	189	4536
	Chip3	127,437	58,993	46.3	54,364	92.2	23	189	4347
	Chip4	149,983	71,883	47.9	66,338	92.3	25	190	4750
	Chip5	152,618	72,098	47.2	67,405	93.5	26	190	4940
	Chip6	147,409	67,605	45.9	62,696	92.7	25	190	4750
	Chip7	142,459	67,231	47.2	61,420	91.4	26	190	4940
	Chip8	145,938	68,795	47.1	62,701	91.1	26	190	4940
**Total**		**1,163,812**	**545,862**		**504,418**				
**Average (per chip)**		**145,477**	**68,233**	**46.9**	**63,052**	**92.4**			
**Grand Total**		**2,377,472**	**1,129,057**		**1,040,400**				
**Average (per chip)**		**148,592**	**70,566**	**47.5**	**65,025**	**92.2**			
**Expected (per chip)**		**177,000**	**88,500**	**50**	**88,500**				
**Performance (%)**		**84**	**80**	**95**	**73**				

Supplementary Table 1: Droplet pairing and filtering statistics for rounds 1 and 2 of human associated virus panel testing.

Gene	Mutation
Reverse transcriptase	K65R
Reverse transcriptase	K103N
Reverse transcriptase	V106M
Reverse transcriptase	Y181C
Reverse transcriptase	M184V
Reverse transcriptase	G190A
Integrase	66A
Integrase	66I
Integrase	66K
Integrase	74M
Integrase	92G
Integrase	92Q
Integrase	97A
Integrase	121Y
Integrase	138A
Integrase	138K
Integrase	140A
Integrase	140S
Integrase	143C
Integrase	143H
Integrase	143R
Integrase	147G
Integrase	148H
Integrase	148K
Integrase	148R
Integrase	155H
Integrase	263K

Supplementary Table 2. List of HIV drug-resistance mutations tested in this study.

Supplementary Discussion

1. The sensitivity of CARMEN is equivalent to that of SHERLOCK: In addition to the data presented in Fig. 1c and Extended Data Fig. 3, there is no “first principles” reason to expect that CARMEN would have lower sensitivity than SHERLOCK, to our knowledge. The chemical composition of the Cas13 detection reactions are very similar between CARMEN and SHERLOCK. The only differences are the concentration of the cleavage reporter, which is 4× higher for CARMEN than for SHERLOCK, and the overall volume of each detection reaction, which is ~10,000 times lower for CARMEN than SHERLOCK. It should also be noted that both approaches start with samples that have been amplified, therefore the number of input molecules into the detection reactions is not limiting, even for small-volume detection reactions. Notably, at the limits of CARMEN’s sensitivity (1 copy per microliter), the user must use a sufficient volume of sample as input to the amplification reaction to ensure that multiple copies of viral nucleic acid are present in the amplification reaction.

Regarding the types of amplification used in CARMEN and SHERLOCK: SHERLOCK was developed with RPA, and CARMEN has been demonstrated with both RPA and PCR. While the amplification approaches may vary, as long as the amplification portion of CARMEN is reasonably efficient, single-molecule sensitivity can readily be achieved. This is analogous to various multiplexed PCR approaches (e.g., BioFire panels).

2. The specificity of CARMEN is derived from Cas13-crRNAs, not primers: Many PCR-based nucleic acid detection methods require highly specific amplification primers because the amplification itself is the detection: any off-target amplification confounds on-target signal. CARMEN benefits from the sensitivity of amplification without the pitfalls of off-target amplification because of the exquisite specificity of the Cas13 enzyme. The Cas13-crRNA complex only recognizes sequences that are complementary to the crRNA, regardless of off-target amplification. Additionally, the specificity of crRNA-guided Cas13 detection is governed by an enzyme recognition event that is far more specific than the thermodynamics of nucleic acid hybridization, which governs primer specificity. Cas13-crRNA can thus detect even single nucleotide differences between sequences. Furthermore, as the specificity of each assay (amplification primers and crRNA) is mainly derived from the crRNA specificity, constraints on primer specificity can be relaxed to capture more sequence diversity in a smaller number of amplification reactions.

3. Flexibility of experimental design: The number of tests in each CARMEN-Cas13 assay is the product of the number of samples (M) and the number of detection mixes (N), which can be determined by the needs of a user (e.g. 10 samples × 100 detection mixes, or 100 samples × 10 detection mixes) (Extended Data Fig. 3). Notably, CARMEN shines in cases when the test matrix is approximately square: the number of samples and detection mixes are both high (e.g. >10). To perform such an experiment conventionally, liquid handling (whether manual or robotic) is complex and time-consuming, reagent consumption is costly (see cost analysis below), and testing may be limited by the available sample quantity. CARMEN circumvents these issues using miniaturization and droplet self-organization (see main text). For use-cases where high sample

throughput alone is desired (many samples × 1 detection mix), CARMEN dramatically reduces costs (see below), but the experiment setup is linear (samples × 1), so a multichannel pipet is equally time-efficient. For use-cases where multiplexed detection alone is desired (1 sample × many detection mixes), the user may consider metagenomic sequencing if the sensitivity is sufficient for the application, while CARMEN may be ideal in cases where exquisite sensitivity and extensive multiplexing are both required.

4. Microwell array statistics: The number of tests that can be performed on one chip depends on the number of productive droplet pairs per chip and the number of replicates per test that are required to make an accurate call.

4.A. Factors affecting the number of productive droplet pairs per chip: The microwell array of a standard chip contains ~42,000 microwells. By empirical observation, loading efficiency is ~75%, and an additional ~10% of microwells are lost to color code filtering (see below). Finally, stochastic droplet pairing produces ~50% productive droplet pairs (one droplet containing amplified sample and one droplet containing detection mix). Overall, ~12,000-16,000 droplet pairs produce useful data per chip. The mChip microwell array contains ~177,000 microwells, resulting in ~65,000 useful droplet pairs/chip (Supplementary Table 2).

4.B. Factors affecting the number of replicates per test required to make an accurate call: The vast majority of positive detection reactions have high signal above background and little replicate-to-replicate variability, and color code classification is very good (>99.5% accuracy after filtering, see Extended Data Fig. 4), suggesting that the number of requisite replicates per test could be quite low. As an experimental measure of the number of replicates needed to correctly identify signal above background, bootstrap analysis was performed on CARMEN-Cas13 Zika detection data (Extended Data Fig. 3 and and Methods, 'Analysis of Zika detection' under 'Zika detection'), revealing a minimum of 3 replicates to correctly call signal above background in >99.9% of bootstrap samples.

It should be noted that the number of replicates required to make an accurate call varies by application type. For nucleic acid detection, which is a near-binary readout, 3 replicates is sufficient. However, for SNP discrimination, which relies on differentiating the relative reaction rates of two crRNAs with a given target, bootstrap analysis suggests that 10-15 replicates are necessary (data not shown). Additionally, for quantitative applications (Extended Data Fig. 9c and d), many replicates may be necessary to yield a result within a desired tolerance (e.g. 5%) of the ground truth value.

4.C. How to calculate the number of tests that can be performed on one chip using the values determined above. Droplet pairing in the microwell array is stochastic; thus, the distribution of the number of replicates per test is Poisson. The user can set the average number of replicates per test (the average of the Poisson distribution) higher or lower to control the probability of test dropout due to under-sampling. For example, using an average of 12 replicates per test, the probability of any test being uninterpretable because of a lack of replicates (<3 replicates) is 1 in 2,000. For a standard chip (~12,000 productive droplet pairs), an average of 12 replicates per test

permits 1,000 tests per chip with a dropout rate well below 1 per chip (1 in 2000). For mChip, which yields ~65,000 droplet pairs, performing 5,000 tests per chip results in an average of 14 replicates per test and reduces the probability of dropout to 1 in 10,000 (below 1 per chip). In situations where delivering a result for every test is essential, such as clinical diagnostics, the average replicate level can be further increased to ensure that sampling for every test is high and the dropout rate due to under-sampling is vanishingly low.

4.D. False negatives and false positives due to color code misclassification: If enough replicates of a test are misclassified, the outcome of the test could change. The fluorescence value of a test is the median value of all replicates; for the median of a positive test to drop to background (i.e. become a false negative), the majority of the replicates would have to be misclassified droplet pairs with no signal above background (dark droplet pairs). Since the detection matrix is sparse, the odds of a misclassified droplet pair being a dark droplet pair are high (99% in the human-associated virus panel testing). This dramatically increases the odds of false negatives compared to false positives. For false negatives, assuming a droplet misclassification rate of 0.005 (see Extended Data Fig. 4), the probability of an individual droplet pair being misclassified is 0.01. With 5 replicates, the odds of the majority of replicates being misclassified is $0.01 \times 0.01 \times 0.01 \times$ (5 choose 3) = 1 in 100,000. Increasing to 7 replicates reduces the chance to <1 in 2 million. Thus, in situations where ensuring accurate calls is critical, such as clinical diagnostics, the number of replicates may be increased modestly to dramatically decrease the odds of a miscalled test due to droplet misclassification.

5. Fidelity of color code analysis

5.A. Controlling exchange of solutes between droplets during pooling: The kinetics of small molecule exchange in the droplet-microwell platform have been described previously[8]. Small molecules may partition into surfactant micelles and exchange between droplets during the pooling step, which lasts <10 min. The exchange of fluorescent dyes during pooling is negligible and does not compromise color code classification[8]. Once droplets are loaded into the microwell array, the Parylene-coated walls of the PDMS microwells prevent further exchange[8]. Diffusion of larger hydrophilic or charged molecules is not a concern in our system since the surfactant-dependent mechanisms by which small molecules can exit droplets are neither expected nor observed to enable protein or nucleic acid escape. Indeed, commercially available systems for ultra-sensitive nucleic acid detection based on similar oils, surfactants, and buffers (e.g. digital droplet PCR) are well-established.

5.B. Color code analysis: Color code classification is robust (Extended Data Fig. 4). After creating and characterizing a set of color codes, the codes are used out-of-the-fridge for each experiment with no additional calibration. Normalizing each color code to the sum of the three fluorescent dyes comprising the 3-color space (Alexa Fluors 647, 594, and 555) makes the system robust to fluorescence imaging artifacts, and discrete color code clusters readily appear. Each cluster represents a droplet set with known contents (e.g. droplets from detection mix 4). Indeterminate points in color space are filtered out by introducing a threshold for the maximum distance a droplet's color code can be from the center of its color code cluster (i.e. a distance threshold, see

Methods, ‘Data analysis’ under ‘General procedures,’ and Extended Data Fig. 4). In the rare case where one color code cluster begins to overlap another, only the two clashing clusters are impacted (and can almost always be resolved, albeit with a loss of replicates), leaving the rest of the color codes unaffected. Such clashing color codes may be omitted from future experiments without any detrimental effect on the set as a whole, and the user does not have to recreate the entire color code set. While particle-based color codes boast higher precision[1,2] than the solution-based color codes presented in this work, the ease of sourcing commercial dyes for solution-based codes may better serve the needs of users who do not require the highest precision or particle-immobilization of assay components.

6. Cost and sample consumption analysis: A key advantage of CARMEN-Cas13 is that it miniaturizes Cas13 detection reactions, thereby reducing reagent and sample consumption per test. Reagent and consumables costs dominate when testing dozens of samples against hundreds of targets using conventional large-volume (tens of microliters) assays, such as SHERLOCK, DETECTR, qPCR, ELISA, and LAMP. Thus, we sought to quantify the cost advantage conferred by CARMEN over these methods when testing many samples against many targets.

6.A. Reagent costs: To analyze the costs associated with CARMEN-Cas13, we first considered the cost of detection reagents alone, and then considered additional costs (plastics including arrays, droplet generation, and color codes). CARMEN-Cas13 typically reduces detection volumes by >400-fold per test, (from 92 µl to perform 4 replicates of a standard 20 µl detection reaction to less than 0.2 µl to perform a CARMEN-Cas13 test with an average of 10 replicate droplet pairs). This results in a >300-fold reduction in cost relative to SHERLOCK, as we use a 4× higher concentration of the fluorescent cleavage reporter in CARMEN-Cas13 (see Extended Data Table 1). Accounting for an additional fixed cost per chip and the cost of color coding and emulsifying samples, the cost per test for CARMEN-Cas13 is >100-fold cheaper than the equivalent SHERLOCK test (see Extended Data Table 1).

Extraction kits are not included in our cost estimates, as they are required for all nucleic acid tests (e.g., RT-qPCR, NGS, PCR + Sanger sequencing) and do not differentiate costs across approaches. Our reagent cost estimates include:

- PCR reagents - included in “marginal cost per sample” line
- Cas13 and crRNAs - included in “marginal cost per detection mix” line as part of “detection reagents”
- Cleavage reporter (RNase Alert v2) - included in “marginal cost per detection mix” line as part of “detection reagents”
- T7 RNA polymerase - included in “marginal cost per detection mix” line as part of “detection reagents”
- RNase inhibitors - included in “marginal cost per detection mix” line as part of “detection reagents”

6.B. Equipment costs: Equipment costs for CARMEN are high, but are not dramatically higher than other multiplexed methods for nucleic acid detection and could be improved in the future. Like many other methods using a fluorescent readout (qPCR, FISH), CARMEN-Cas13 requires sensitive detection of fluorescence in 4-5 channels. CARMEN-Cas13 also requires some automated imaging capabilities to facilitate data acquisition from the microwell array. Multimode plate readers or qPCR machines cost about $30,000, whereas a microscope suitable for CARMEN costs about $50,000 (the additional cost coming from the imaging requirements for CARMEN). Both of these are much cheaper than Illumina sequencing machines typically used for high-throughput metagenomic sequencing (e.g. HiSeq, NextSeq, NovaSeq).

In addition to equipment for fluorescent readout, CARMEN also requires equipment for droplet generation. While a commercial machine, the Bio-Rad QX200 ($31,000), can be used for droplet generation, the equipment requirements for droplet generation can be substantially reduced by using a custom-fabricated pressure manifold, which costs approximately $2,000 to make[8]. Thus, droplet generation hardware is a minor component of the CARMEN technology's overall cost.

6.C. Labor costs: While labor costs are difficult to quantify, the amount of labor required for CARMEN-Cas13 is lower per test than for low-plex assays like RT-qPCR, ELISAs, or LAMP. Although it takes, for example, ~7 person-hours to set up, image, and analyze an individual mChip, the ~5,000 tests per chip is equivalent to >50 full 384-well plates (containing 3-4 technical replicates per test, the number necessary to achieve statistical power in plate-based assays). Thus, the time required per full 384-well plate equivalent is <10 person-minutes; in our hands, setting up one full 384-well plate takes at least an hour; starting with thawed reagents and ending at the start of the assay. In addition, the protocol for CARMEN-Cas13 is simpler than library preparation for next-generation sequencing, requiring fewer steps and less time to complete.

6.D. Cost scaling: It should be noted that the scale of the experiment is important to consider when comparing the costs of performing CARMEN-Cas13 relative to other assays. In particular, many of the associated costs scale with the number of chips, or linearly with the sum of the number of amplified samples and the number of Cas13 detection mixes. As such, a less favorable use case for CARMEN-Cas13 would be testing 1 sample for hundreds of potential viruses: due to the fixed costs, the cost savings will be smaller relative to performing the same experiment in a standard microtiter plate. The cost drops substantially when multiple samples are tested simultaneously, as the marginal cost of adding a new sample to a particular chip is only a few dollars. The combinatorial nature of CARMEN further reduces the cost of testing many samples for the presence of many targets. It should be noted that in the limit of low reagent cost per test, sample processing will likely dominate total cost, as sample costs scale with the number of samples rather than the number of tests being performed. Thus, to enable sample testing at even higher throughput than CARMEN-Cas13, one would need to significantly reduce the cost and labor associated with sample collection and processing.

6.E. Sample volume requirements: Performing dozens or hundreds of SHERLOCK, DETECTR, qPCR, ELISA, or LAMP assays on a patient sample requires a very large sample volume (tens of milliliters of blood, saliva, or urine), which is often not available. For CARMEN, at most 2

microliters of cDNA made from extracted RNA are used per PCR pool, for a total of up to 30 microliters for 15 PCR pools in the human-associated viral panel. This requires a total sample input volume of a few hundred microliters of bodily fluid (depending on the type of extraction kit used). In short, the overall input sample volume requirements for CARMEN do not vary substantially from other methods, despite a considerable increase in the number of tests performed on each sample. Thus, in addition to reducing reagent costs, CARMEN-Cas13 reduces sample consumption, thereby enabling more tests to be run and reducing sample acquisition and processing costs.

7. CARMEN workflow time: The workflow for CARMEN significantly reduces the assay time and labor necessary for assembling detection reactions in cases where multiple samples are being tested for multiple nucleic acid sequences. The workflow can be divided into two key steps:

1. Preparation of amplified samples and detection reagents, which is typically completed in 2-3 hours
2. Use of the droplet-microwell array to test each sample, which consists of 2-3 hours of hands-on time and a 1-hour incubation.

The entire CARMEN workflow, from sample preparation to data analysis, takes <7 hours and can be completed by one researcher in one workday.

As an illustrative example, we outline the time necessary to test 40 samples for 100 nucleic acid sequences (4,000 tests; with an average of 13 replicates per test, this totals 52,000 detection reactions).

1. 3 hours: preparation of amplified samples and detection reactions, including PCR reaction and dilutions (Extended Data Fig. 2, Step 1).
2. 1 hour: Color coding and emulsification using a Bio Rad droplet generator takes 2.5 min per 8 samples or 8 detection mixes (Extended Data Fig. 2, Step 1).
3. 10 minutes: Droplet pooling is rapid, requiring <30 seconds per 8 samples (using a multichannel pipet) (Extended Data Fig. 2, Step 2).
4. 15 minutes: The chip is loaded in a single pipetting step, followed by a series of oil washes, and chip sealing (Extended Data Fig. 2, Steps 3-4).
5. 18 minutes: Color code imaging with 4-color color codes on an mChip using $1\times$ magnification takes ~18 minutes (Extended Data Fig. 2, Step 4). A standard size chip requires ~8 minutes of imaging time.
6. < 1 minute: Merging is nearly instantaneous (Extended Data Fig. 2, Step 5).
7. 1 hour: Optimal reaction incubation time varies based on crRNA sensitivity, viral titre, and application type; in the vast majority of cases, including the HAV panel, we use a 1 hour incubation (Extended Data Fig. 2, Step 5). The range of incubation times used in this work is 0.5-3 hours.
8. 18 minutes: Fluorescence reporter imaging (Extended Data Fig. 2, Step 6).
9. 30 minutes: Data analysis using the existing package is extremely straightforward: color code assignment can be performed while the assay is running (~20 min), so only the final images need to be analyzed after the assay is complete (<20 min, followed by graphical plotting).

Thus, the entire workflow, from PCR to results, takes <7 hours.

Notably, the protocol for CARMEN is far simpler and shorter than the protocol for metagenomic sequencing directly from patient samples. Unlike many genome sequencing protocols, which start with microgram quantities of DNA, patient blood, urine, or saliva samples only contain nanograms of nucleic acid, the vast majority of which is host genetic material[4]. The resulting sequencing library construction therefore requires many enzymatic reactions after first strand synthesis (RNase H treatment, second strand synthesis, ribosomal RNA depletion, nuclease treatments, nucleic acid fragmentation, ligation), with purification steps required in between each step[3]. Overall, these protocols require multiple days to complete, followed by complex data analysis that requires expert interpretation[4]. In contrast, CARMEN requires setting up a single set of amplification reactions, followed by Cas13 detection reactions, with no sample purification required in between, and data analysis can be performed in a few steps from a Jupyter notebook.

While microarrays are not as difficult to prepare as sequencing libraries, there are still multiple enzyme reactions and purification steps required, as well as overnight hybridization. This means that the turnaround time for microarrays is still multiple days. Furthermore, microarrays do not provide the specificity of CRISPR-based detection or sequencing.

Finally, we want to reiterate that CARMEN is vastly more efficient in terms of workflow than traditional SHERLOCK. For example, to perform 4,000 test using traditional SHERLOCK, 42 × 96-well plates would be required: an aggressive estimate of 2 hours to prepare each plate would require >80 hours for plate preparation alone, not including plate reading and data analysis. Thus, CARMEN can reduce weeks of labor to just a single day.

8. Reduction in liquid handling steps using CARMEN: The ability of the droplet-microwell array to reduce liquid handling has been documented previously[8]. As a relevant example in this context, we calculate the number of liquid handling steps required to construct a 40 sample × 100 detection mix assay with at least 3 replicates per test (12,000 reactions total) using CARMEN and standard liquid handling.

Standard liquid handling:

1. 4,000 reactions × 3 replicates = 12,000 steps for addition of sample
2. 4,000 reactions × 3 replicates = 12,000 steps for addition of detection mix
3. Total: 24,000 steps

CARMEN:

1. 140 steps (18 steps with multichannel pipet): add color codes to a fresh plate
2. 140 steps (18 steps with multichannel): add samples and detection reagents to color codes
3. 140 steps (18 steps with multichannel): add color coded samples and detection reagents to droplet generator cartridges
4. 140 steps (18 steps with multichannel): add oil to droplet generator cartridges
5. 140 steps (18 steps with multichannel): transfer droplets to fresh plate
6. 140 steps (18 steps with multichannel): pool droplets to one row of a plate

7. 7 steps: pool the wells of the plate into one tube
8. 1 step: load chip
9. Total: 848 steps (116 steps with a multichannel)

Modern liquid handlers are the result of extensive commercial engineering efforts to simplify the user experience. In a similarly commercialized CARMEN platform, we envision that the user could add their samples and detection reagents to an arrayed droplet generator cartridge that automatically pools droplets. In the commercialized platform the user liquid handling steps would be:

1. 140 steps (18 steps with multichannel): add samples and detection reagents to droplet generator cartridges
2. 1 step: load chip
3. Total: 141 steps (19 steps with a multichannel pipet)

Finally, the practical considerations of volume and time should also be considered when comparing CARMEN to automated liquid handling. These aspects are detailed below:

Volume required for automated liquid handling: Using automated liquid handling, the reactions would be assembled in a multiwell plate, where evaporation over the 1-hour incubation time would be a concern. We expect that volumes of at least 1 μl would be needed to mitigate evaporation concerns. This raises sample consumption and the costs of the detection mix 1,000× compared to CARMEN (which uses 1 nl volumes without concern about evaporation).

Time required for automated liquid handling: Furthermore, converting the number of steps into the time necessary to construct 12,000 reactions using automated liquid handling reveals that CARMEN is faster than automated liquid handlers. To the best of our knowledge, the Labcyte Echo is the state-of-the-art for rapid liquid handling with the ability to rearray reagents (every combination of sample and detection mix is required, so entire rows, columns, or plates cannot be transferred at once). The fastest Labcyte that transfers 1 μl volumes is the Echo 525, and the manufacturer advertises the transfer of a 384W plate into a fresh 384W plate in 3.3 min (~1.9 liquid handling steps per second), see https://www.labcyte.com/media/pdf/SPC-Echo-525-Liquid-Handler.pdf. We believe that 384W plates are likely the format that would be used in such an experiment, since the PCR reactions and detection mixes would be added to the source plates manually using a multichannel pipette. Using the manufacturer's estimate of 1.9 liquid handling steps per second, the Echo 525 would require 3.5 hours to assemble the requisite 12,000 reactions, compared to <2 hours using CARMEN (see time analysis in Supplementary Discussion Section 9), or minutes using a fully commercialized version of CARMEN. Moreover, if the reactions were set up using an automated liquid handler, additional time would be necessary to read the reaction outputs, and additional logistical hurdles, including timing of reagent preparation, plate assembly, reaction initiation, plate exchanges, plate initialization, and assay readout, would need to be addressed. Using CARMEN, the reactions start simultaneously when droplets are merged across the chip, and the fluorescence output of all reactions is read in <18 min on a microscope.

9. Human-associated viral panel

9.A. Selection of optimal crRNAs for testing: Due to the high cost of synthesizing hundreds of synthetic DNA and RNA oligonucleotides, we did not test the entirety of our human-associated viral panel design experimentally. The vast majority (143) of species required a single crRNA to cover 90% of known sequences (Extended Data Fig. 5k), thus we decided to test a single crRNA for each species. In cases where there were multiple crRNAs in a set, the crRNA whose sequence most closely matched the majority consensus sequence for the species was chosen. Based on our results using crRNA sets for sub-subtyping of influenza A (Extended Data Fig. 8), it is likely that one could use the complete crRNA sets to fully cover 90% of the known sequences in each species, as designed. Our barcode and multiplexing scheme would be able to accommodate this, with a moderate decrease in sample throughput due to the increased number of detection mixes.

9.B. Cross-contamination: A practical concern of testing a massively multiplexed viral detection panel is cross-contamination, especially pre-emulsification. The extreme sensitivity of the CARMEN-Cas13 system means that even trace cross-contamination could lead to widespread false-positive results. Widespread cross-reactivity was not observed during our testing, however there were some examples of cross-reactivity between a crRNA and an unexpected synthetic target. All examples of cross-reactivity were investigated by aligning crRNA and synthetic target sequences. Based on this analysis, a handful (4-5) of these examples were likely sequence-mediated, and were modified in the version 2 redesign. The remaining examples of cross-reactivity are likely due to cross-contamination for the following reasons:

1. The vast majority of cross-reactivity that was not sequence-mediated occurred between neighboring wells, suggesting that it could be due to cross-contamination during the dilution of synthetic targets, or during the setup of amplification reactions.
2. It is possible that the cross-reactivity is due to cross-contamination that occurred during DNA or RNA synthesis. The oligonucleotides for the human-associated virus panel were synthesized commercially, in parallel, in 96-well plates. Co-synthesized oligonucleotides used as barcoded adapters for next-generation sequencing have been observed to have cross-contamination at low frequencies[5].
3. It is possible that this unexpected reactivity in the healthy control samples may represent true infection in the healthy controls or contamination from the laboratory environment, the results from these crRNAs are uninterpretable for the specified round of testing, as is common custom in diagnostic testing.

As is commonly observed in highly multiplexed diagnostics, both CARMEN and NGS identified unexpected and unconfirmed infections in a small fraction of the samples, which may represent contamination from the laboratory environment or real infections (Fig. 2i). The presence of a low level of contamination in our lab detected in the negative controls, and seen in CARMEN, PCR, and RNA sequencing, is a result of our laboratory set up in the two infectious disease research labs where this work was performed. The workspaces used to generate results presented here were *not* CLIA-certified clinical testing lab, and instead are research labs studying numerous infectious diseases in small shared spaces. It is true that all technologies that involve amplification

will have challenges with contamination, but this issue is generic to nucleic acid amplification tests (NAATs) of all types, including numerous FDA-approved diagnostics. Such routine contamination is overcome by engineering controls and specific protocols in clinical-grade diagnostic laboratories. Relevant to the research and technology development in our lab, we can account for contamination by quality control metrics such that minor contamination does not confound the interpretation of research data.

9.C. Sequence coverage: In addition to cross-reactivity, sequence coverage is an important aspect of design. The human-associated virus panel was designed to cover at least 90% of known sequences for each species, but the actual coverage might be higher or lower for the following reasons.

1. Our crRNAs and primers were designed to cover at least 90% of the known sequences for each species in the panel, but it is possible that they could also detect the 5-10% of known sequences that are not supposed to be covered by design.
2. We set a stringent threshold of 1 mismatch between a crRNA and its target. Depending on the position of the mismatch, there could still be substantial cleavage activity; truncated spacers can be quite active for nucleic acid detection[7].
3. For some species, not have enough sequence data is available to design an accurate diagnostic; thus we restricted our panel to species with ≥10 available genome sequences.

Similar considerations also apply to the influenza subtyping panel.

Finally, sequence coverage and analytical sensitivity are distinct but related considerations that contribute to assay sensitivity: a given crRNA targets a specific sequence within the genome with a certain analytical sensitivity (ability to detect that sequence above background). To increase assay sensitivity, a user may add more crRNAs to be able to detect additional fragments of pathogen nucleic acid (increasing sequence coverage) or improve the performance of individual crRNAs. Multiplexing crRNAs to increase sequence coverage is particularly effective when samples may carry only a portion of the known viral genome (due to degradation, mutation, etc.).

9.D. Testing of unknown samples: In this study, we tested 169 known, synthetic targets with the majority consensus sequence of each of the 169 species in the human-associated viral panel, using a single primer pool to amplify each target (based on the design). For unknown samples, one would amplify each sample with all 15 pools, and then combine the pools prior to detection (into 5 metapools, defined in Methods, 'Nucleic acid amplification' under 'HV panel'), or run them separately. The following outcomes are possible:

1. One may observe selective identification with a single crRNA and rejoice.
2. If one observes cross-reactivity, one can rerun the individual pool where the cross-reactivity occurred. In these cases, one should not assume that there is a co-infection, unless there is prior information suggesting that a co-infection is likely.
3. Weak reactivity may be accounted for by using positive controls or retesting samples to increase the confidence in the result.
4. No positive results may be observed for the following reasons: (1) the sequence of the pathogen is in the 5-10% of known sequences not covered by the design; (2) the viral titers could be too low to detect; or (3) the sample could be degraded.

9.E. Comparing CARMEN and sequencing data: It is important to keep in mind that the CARMEN assays we have designed in this study target a single amplicon in a viral genome, whereas next-generation sequencing can potentially detect the presence or absence of any portion of a viral genome. We have thus chosen to compare the CARMEN and sequencing data in two distinct ways:

1. Presence of the CARMEN amplicon in the sample. Here, we assemble viral genomes using viral-ngs and check to ensure there is coverage across the entire CARMEN amplicon in the assembled genome. This allows for a direct comparison of whether we expect to detect a particular virus in a sample using CARMEN.
2. Presence of any viral sequence in the sample. Here, we use Kraken to assign each read to specific viral taxa (at the species level), with a threshold of at least 1 read per million. This is not an apples-to-apples comparison with CARMEN, but provides useful information about the overall quality of the nucleic acid present in each sample.
3. We tested the original samples for each expected virus using commercially available RT-PCR assays. The results were highly concordant with CARMEN and NGS for viruses with low sequence diversity but variable for highly diverse viruses (HIV and HCV) (Extended Data Fig. 8b).
4. In 1 HIV sample and 2 HCV samples, sequencing detected the crRNA locus but CARMEN did not detect the virus. In the HIV sample, there were 3 mismatches between the crRNA sequence and the sequence of the crRNA locus in the sample. One of the HCV samples had 1 mismatch between the crRNA sequence and the sequence of the crRNA locus in the samples. These mutations may have reduced the activity of these particular crRNAs in these samples.

By designing CARMEN assays with multiple independent amplicons, it should be possible to increase the sensitivity and specificity of CARMEN even further than what we have already demonstrated. Indeed, our HIV RT panel demonstrates this principle, as it contains crRNAs that target three different amplicons in the reverse transcriptase gene. In this study, we primarily used the HIV RT panel for SNP identification, but it can also be used for detection of HIV.

9.F. Detection of unexpected viruses with CARMEN: We detected a few viruses using CARMEN that were not previously known to be in the samples, detailed below:

1. Pegivirus A (Pegi A) is a common coinfection with HIV1[6]. CARMEN identified pegivirus A in the paired serum and plasma samples of one patient, as well as the serum samples from two other patients, with HIV infections (4 samples total). RNA sequencing confirmed the presence of pegivirus nucleic acid in all of these samples.
2. Torque teno-like mini virus (TLMV) was discovered across dengue, Zika, HIV, and HCV samples by CARMEN (30 samples total, all from blood), although never in influenza A samples (throat or nasal swabs), which is consistent with the fact that this virus is commonly found in human blood[7]. RNA sequencing confirmed the presence of the CARMEN-targeted TLMV crRNA in 15/30 samples and the presence of any TLMV nucleic

acid in 21/30 samples. The discovery of TLMV in multiple human samples reaffirms the fact that this is likely true detection of a virus found in blood.

References

1. Nguyen, H. Q. *et al.* Programmable microfluidic synthesis of over one thousand uniquely identifiable spectral codes. *Adv Opt Mater* **5**, (2017).

2. Zhao, Y. *et al.* Microfluidic generation of multifunctional quantum dot barcode particles. *J. Am. Chem. Soc.* **133**, 8790–8793 (2011).

3. S. R. Head *et al*. Library construction for next-generation sequencing: overviews and challenges. *Biotechniques*. **56**, 61-64 (2014).

4. C. Y. Chiu, S. A. Miller, Clinical metagenomics. *Nat. Rev. Genet*. **20**, 341-355 (2019).

5. M. A. Quail *et al.*, SASI-Seq: Sample Assurance Spike-Ins, and highly differentiating 384 barcoding for Illumina sequencing. *BMC Genomics*. **15** (2014), doi:10.1186/1471-2164-15-110.

6. Mohr, E. L. & Stapleton, J. T. GB virus type C interactions with HIV: the role of envelope glycoproteins. *J. Viral Hepat*. **16**, 757–768 (2009).

7. Moen, E. M., Huang, L. & Grinde, B. Molecular epidemiology of TTV-like mini virus in Norway. *Arch. Virol*. **147**, 181–185 (2002).
