# Supplementary material for: Massively multiplexed nucleic acid detection with Cas13
Source: Nature. 2020 Apr 29;582(7811):277–82. doi: 10.1038/s41586-020-2279-8 (PMC7332423; doi:10.1038/s41586-020-2279-8)
Supplement: Supplementary file 2 — Reporting Summary [file 41586_2020_2279_MOESM2_ESM.pdf]

nature research

Corresponding author(s): Cameron Myhrvold and Paul Blainey

Last updated by author(s): February 26, 2020

Reporting Summary

Nature Research wishes to improve the reproducibility of the work that we publish. This form provides structure for consistency and transparency in reporting. For further information on Nature Research policies, see Authors & Referees and the Editorial Policy Checklist.

Statistics

For all statistical analyses, confirm that the following items are present in the figure legend, table legend, main text, or Methods section.

n/a	Confirmed	
☐	☒	The exact sample size (*n*) for each experimental group/condition, given as a discrete number and unit of measurement
☐	☒	A statement on whether measurements were taken from distinct samples or whether the same sample was measured repeatedly
☐	☒	The statistical test(s) used AND whether they are one- or two-sided<br>*Only common tests should be described solely by name; describe more complex techniques in the Methods section.*
☒	☐	A description of all covariates tested
☒	☐	A description of any assumptions or corrections, such as tests of normality and adjustment for multiple comparisons
☐	☒	A full description of the statistical parameters including central tendency (e.g. means) or other basic estimates (e.g. regression coefficient) AND variation (e.g. standard deviation) or associated estimates of uncertainty (e.g. confidence intervals)
☒	☐	For null hypothesis testing, the test statistic (e.g. *F*, *t*, *r*) with confidence intervals, effect sizes, degrees of freedom and *P* value noted<br>*Give P values as exact values whenever suitable.*
☒	☐	For Bayesian analysis, information on the choice of priors and Markov chain Monte Carlo settings
☒	☐	For hierarchical and complex designs, identification of the appropriate level for tests and full reporting of outcomes
☒	☐	Estimates of effect sizes (e.g. Cohen's *d*, Pearson's *r*), indicating how they were calculated

Our web collection on statistics for biologists contains articles on many of the points above.

Software and code

Policy information about availability of computer code

Data collection	Matlab 2013 for microscope and camera control
Data analysis	Python 2.7, custom analysis code available at github akulesa/kchip, mafft v7.31 was used for primer and crRNA sequence design

For manuscripts utilizing custom algorithms or software that are central to the research but not yet described in published literature, software must be made available to editors/reviewers. We strongly encourage code deposition in a community repository (e.g. GitHub). See the Nature Research guidelines for submitting code & software for further information.

Data

Policy information about availability of data

All manuscripts must include a data availability statement. This statement should provide the following information, where applicable:

- Accession codes, unique identifiers, or web links for publicly available datasets
- A list of figures that have associated raw data
- A description of any restrictions on data availability

Viral genome alignments and sequencing data will be made publicly available. Raw data may be made available upon request.

Field-specific reporting

Please select the one below that is the best fit for your research. If you are not sure, read the appropriate sections before making your selection.

☒ Life sciences ☐ Behavioural & social sciences ☐ Ecological, evolutionary & environmental sciences

For a reference copy of the document with all sections, see nature.com/documents/nr-reporting-summary-flat.pdf

Life sciences study design

All studies must disclose on these points even when the disclosure is negative.

Sample size	The number of replicates necessary to make a call was determined by bootstrap, as described in the Supplementary Discussion. Sample sizes were the result of stochastic loading of microwell arrays, as described in the Supplementary Discussion.
Data exclusions	Droplets data were quality filtered for size of droplet (excluding any droplets that may merge across wells or fail to merge) and for the distance between the droplet's color coordinates and those of its assigned color code cluster (excluding droplets where the color code cluster assignment is very uncertain). Exclusions were made based on pre-established quality control filters. Low-quality data were droplets that fell outside of a minimum radius from a cluster, making their cluster assignment ambiguous. By applying a distance filter (illustrated in EDF 5), we remove low-quality data. The cutoff for the distance filter is pre-established based on testing the droplet color codes without samples.
Replication	Each experiment includes at least 3 technical replicates per data point, with a median of 10-15 replicates. Biological replicates were consistent (97% concordance between the replicates of the HAV tests, Ex. Data Fig. 6). Replicates for main text figures are provided in Supplementary File 6.
Randomization	Samples were not randomized into groups, as samples were not grouped.
Blinding	Blinding was performed for the HIV clinical samples for DRM testing, but not for other samples. Blinding was not possible for some samples due to the nature of the patient cohorts selected (they were known to be disease-positive)

Reporting for specific materials, systems and methods

We require information from authors about some types of materials, experimental systems and methods used in many studies. Here, indicate whether each material, system or method listed is relevant to your study. If you are not sure if a list item applies to your research, read the appropriate section before selecting a response.

Materials & experimental systems

n/a	Involved in the study
☒	☐ Antibodies
☒	☐ Eukaryotic cell lines
☒	☐ Palaeontology
☒	☐ Animals and other organisms
☒	☐ Human research participants
☒	☐ Clinical data

Methods

n/a	Involved in the study
☒	☐ ChIP-seq
☒	☐ Flow cytometry
☒	☐ MRI-based neuroimaging
